# The Machado–Joseph Disease Deubiquitinase Ataxin-3 Regulates the Stability and Apoptotic Function of p53

**DOI:** 10.1371/journal.pbio.2000733

**Published:** 2016-11-16

**Authors:** Hongmei Liu, Xiaoling Li, Guozhu Ning, Shu Zhu, Xiaolu Ma, Xiuli Liu, Chunying Liu, Min Huang, Ina Schmitt, Ullrich Wüllner, Yamei Niu, Caixia Guo, Qiang Wang, Tie-Shan Tang

**Affiliations:** 1 State Key Laboratory of Membrane Biology, Institute of Zoology, University of Chinese Academy of Sciences, Chinese Academy of Sciences, Beijing, China; 2 CAS Key Laboratory of Genomics and Precision Medicine, Beijing Institute of Genomics, University of Chinese Academy of Sciences, Chinese Academy of Sciences, Beijing, China; 3 Department of Pathology and Center for Experimental Animal Research, Institute of Basic Medical Sciences, Chinese Academy of Medical Sciences & Peking Union Medical College (PUMC), Beijing, China; 4 University of Bonn, Department of Neurology and German Center for Neurodegenerative Diseases (DZNE), Bonn, Germany; Baylor College of Medicine, United States of America

## Abstract

As a deubiquitinating enzyme (DUB), the physiological substrates of ataxin-3 (ATX-3) remain elusive, which limits our understanding of its normal cellular function and that of pathogenic mechanism of spinocerebellar ataxia type 3 (SCA3). Here, we identify p53 to be a novel substrate of ATX-3. ATX-3 binds to native and polyubiquitinated p53 and deubiquitinates and stabilizes p53 by repressing its degradation through the ubiquitin (Ub)-proteasome pathway. ATX-3 deletion destabilizes p53, resulting in deficiency of p53 activity and functions, whereas ectopic expression of ATX-3 induces selective transcription/expression of p53 target genes and promotes p53-dependent apoptosis in both mammalian cells and the central nervous system of zebrafish. Furthermore, the polyglutamine (polyQ)-expanded ATX-3 retains enhanced interaction and deubiquitination catalytic activity to p53 and causes more severe p53-dependent neurodegeneration in zebrafish brains and in the substantia nigra pars compacta (SNpc) or striatum of a transgenic SCA3 mouse model. Our findings identify a novel molecular link between ATX-3 and p53-mediated cell death and provide an explanation for the direct involvement of p53 in SCA3 disease pathogenesis.

## Author Summary

Ataxin-3 (ATX-3) is a ubiquitously expressed protein that mutated in a neurodegenerative disease called spinocerebellar ataxia type 3 (SCA3). It contains a polyglutamine (polyQ) tract near its C-terminus, the expansion of which is known to be the causative factor for SCA3. It has been known for a long time that ATX-3 is a deubiquitinating enzyme (DUB). However, the substrates targeted by ATX-3 in the physiological context remain elusive, thus largely limiting our understanding of its cellular function and that of the pathogenic mechanism of SCA3. This study has identified p53 to be a novel substrate of ATX-3, and its function is tightly regulated by ATX-3. PolyQ expansion augments ATX-3’s cellular function in p53 regulation. Due to enhanced interaction to p53 and up-regulation of p53, polyQ-expanded ATX-3 led to an increased p53-dependent neuronal cell death in zebrafish and mouse models, thus providing clear in vivo evidences for the direct involvement of p53 in SCA3 pathology. This study not only establishes a basic function of ATX-3 but also provides an explanation of how the interplays between ATX-3 and p53 contribute to the SCA3 pathogenesis; thus, it is an important contribution for the future development of therapeutic approaches for this currently untreatable neurodegenerative disease.

## Introduction

Spinocerebellar ataxia type 3 (SCA3), also known as Machado–Joseph disease (MJD), is an autosomal-dominantly inherited ataxia and one of at least nine polyglutamine (polyQ) neurodegenerative disorders described so far [1–3]. SCA3 is caused by an unstable cytosine-adenine-guanine (CAG) trinucleotide expansion mutation in the *ATXN3* gene leading to an expanded polyQ tract within the ataxin-3 (ATX-3) protein [4]. As a deubiquitylase, ATX-3 is highly conserved and ubiquitously expressed in cells throughout the body [5]. ATX-3 knockout (KO) mice have no major abnormalities [6]. It is possible that, besides ATX-3, three other members of the MJD family of cysteine proteases, including ATX-3 Like, JosD1, and JosD2 [7], may exert similar functions to ATX-3 and compensate for its absence in KO models. ATX-3 has a structured N-terminal Josephin domain comprising the catalytic site, two ubiquitin (Ub)-binding sites, and an unstructured C-terminal, which contains two or three Ub-interacting motifs (UIMs) flanking a polyQ tract [8,9]. The expansion of the polyQ tract is thought to trigger a pathogenic cascade, leading to cellular dysfunction and selective neuronal cell death [10]. Expansion length is inversely correlated with age of disease onset and directly with disease severity. However, the precise pathogenic mechanism triggered by polyQ-expanded ATX-3 in SCA3 patients has remained elusive [11–16].

A number of works have been carried out to explore ATX-3’s biological and potential cellular roles, and identification of molecular partners interacting with ATX-3 is hoped to facilitate identification of its physiological functions. For example, as a highly specialized deubiquitinating enzyme (DUB), a function of ATX-3 has been shown to be involved in the cellular protein quality control system by interacting with p97/valosin-containing protein (VCP) [9,17–22] and several E3 Ub ligases [15,20,23–28]. Moreover, several lines of evidence have shown that ATX-3 can bind DNA and interact with transcription regulators, thus being involved in transcriptional regulation [29–31]. Thus, ATX-3 has been associated with a wide range of biological activities.

The absence of ATX-3 leads to an increase of total ubiquitinated protein levels in ATX-3 KO mice [6], whereas overexpression of ATX-3 results in significantly reduced cellular protein ubiquitination in HEK293 cells [32], suggesting that ATX-3 may regulate the ubiquitination status of many proteins. However, the substrates targeted by ATX-3 in the physiological context remain unclear, thus limiting our understanding of its cellular functions. Whether the polyQ expansion in ATX-3 may contribute to the neuropathology by affecting its molecular interactions with other proteins or endogenous functions of normal ATX-3 is unknown. To develop effective therapies for this incurable disorder, it is important to identify ATX-3’s preferred substrates and to determine how the polyQ expansion causes the protein’s dysfunction.

In the present study, we used immunoprecipitation coupled with mass spectrometry to search for the proteins that associate with ATX-3. We have found that ATX-3 interacts with p53 and functions as a novel p53 DUB. ATX-3 deubiquitinates, stabilizes p53, and further regulates the functions of p53 in transactivation and apoptosis both in vitro and in vivo. Whether and how the polyQ expansion in ATX-3 affects its functional regulation of p53 and further neurodegeneration have also been investigated.

## Results

### ATX-3 Interacts with p53

**Fig 1:**
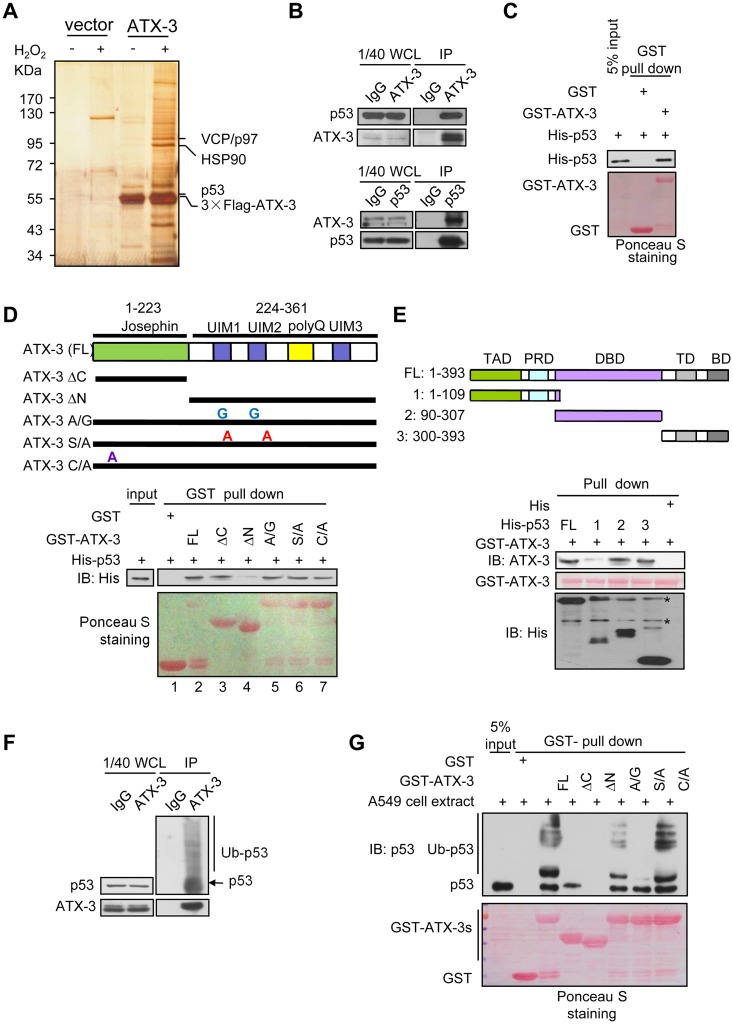
ATX-3 interacts with p53. (A) Silver-stained gel of proteins co-immunoprecipitated from 293T cells transfected with 3×Flag-ATX-3 or control 3×Flag empty vector that were either untreated or treated with H_2_O_2_. Specific ATX-3-associated proteins were identified by mass spectrometry. (B) 293T cell lysates were subjected to immunoprecipitation with control IgG, anti-ATX-3 (upper), or anti-p53 (lower) antibodies and subsequently immunoblotted with indicated antibodies. WCL, whole cell lysate. (C) GST-pull-down assay was performed with the indicated GST-fusion proteins and bacterially expressed His-tagged p53. GST proteins were stained by Ponceau S, and the pulled-down proteins were immunoblotted with anti-His antibody. (D) (Upper) A schematic diagram of full-length (FL) and various mutants of ATX-3. (Lower) His-p53 was incubated with GST or GST-ATX-3 and its mutants coupled to Glutathione-agarose. Pull-downs were stained by Ponceau S, and the presence of bound p53 was assessed by immunoblotting with anti-His antibody. (E) (Upper) A schematic diagram of FL and various p53 protein deletion mutants. (Lower) GST-ATX-3 was incubated with His-p53 (FL and truncated fragments, indicated by arrowheads) coupled to nickel-nitrilotriacetic acid (Ni-NTA) beads. The presence of bound ATX-3 was assessed by immunoblotting with anti-ATX-3 antibody. GST-ATX-3 was stained by Ponceau S. Asterisk indicates nonspecific bands. (F) ATX-3 interacts with ubiquitinated p53 in vivo. Western blot analysis of control immunoprecipitates with IgG or immunoprecipitates with the anti-ATX-3 antibody from 293T cells. (G) GST-pull-down assay was performed with GST or the indicated GST-fusion proteins and the whole cell extract of A549. GST proteins were stained by Ponceau S, and the pulled-down proteins were analyzed by immunoblotting with anti-p53 polyclonal antibody.

We analyzed proteins co-immunoprecipitated with 3×Flag-tagged ATX-3 from H_2_O_2_-treated 293T cells using mass spectrometric analysis (Fig 1A). p53 was found to associate with ATX-3. The interaction between ATX-3 and p53 was confirmed under physiological condition (Fig 1B) and with in vitro purified forms (Fig 1C), indicating a direct association between these two proteins. Furthermore, the amino terminus of ATX-3 was found to be necessary for binding with p53 (Fig 1D). This result was corroborated in cells by immunoprecipitation (S1A Fig). In addition, glutathione S-transferase (GST)–pull-down assay showed that both the DNA-binding domain and the C-terminal regulatory domain of p53 were sufficient for its interaction with ATX-3 (Fig 1E).

The two or three UIMs of ATX-3, depending on the splice isoform, mediate its binding to poly-ubiquitinated substrates. We observed that full-length (FL) ATX-3 bound robustly to both the native and ubiquitinated form of p53 in vitro (S1B Fig) and in cells (Fig 1F). Mutating the active site cysteine 14 did not affect the Ub chain binding activity of ATX-3, whereas ΔC and ΔN deletion as well as the UIM mutations (S236/256A and A232/252G) resulted in either abolished or impaired Ub binding activity (S1D Fig). Consistently, as shown in Fig 1G, the cysteine 14 mutation did not affect the binding of ATX-3 to either native or ubiquitinated p53, whereas the ΔN mutant lost its binding to both forms of p53. The ΔC mutant was found to bind to the native p53 with decreased affinity, and the two UIM mutants showed significantly compromised binding to ubiquitinated p53. We constructed a catalytic inactive ΔC mutant to exclude the possibility that the N-terminal domain might be able to interact with ubiquitinated p53 but further be deubiquitinated before detection. Catalytic inactive ΔC mutant showed similar binding affinity to native p53 as the ΔC mutant did, confirming that N-terminal domain only bound to native p53 (S1C Fig). Taken together, these results indicated that the binding of ATX-3 to p53 was synergistically regulated by the Josephin domain and the UIMs, with the former being primarily responsible for the binding of ATX-3 to the native p53 and further facilitating the latter to bind to ubiquitinated p53.

### ATX-3 Suppresses p53 Ubiquitination in Cells and Deubiquitinates p53 Directly In Vitro

**Fig 2:**
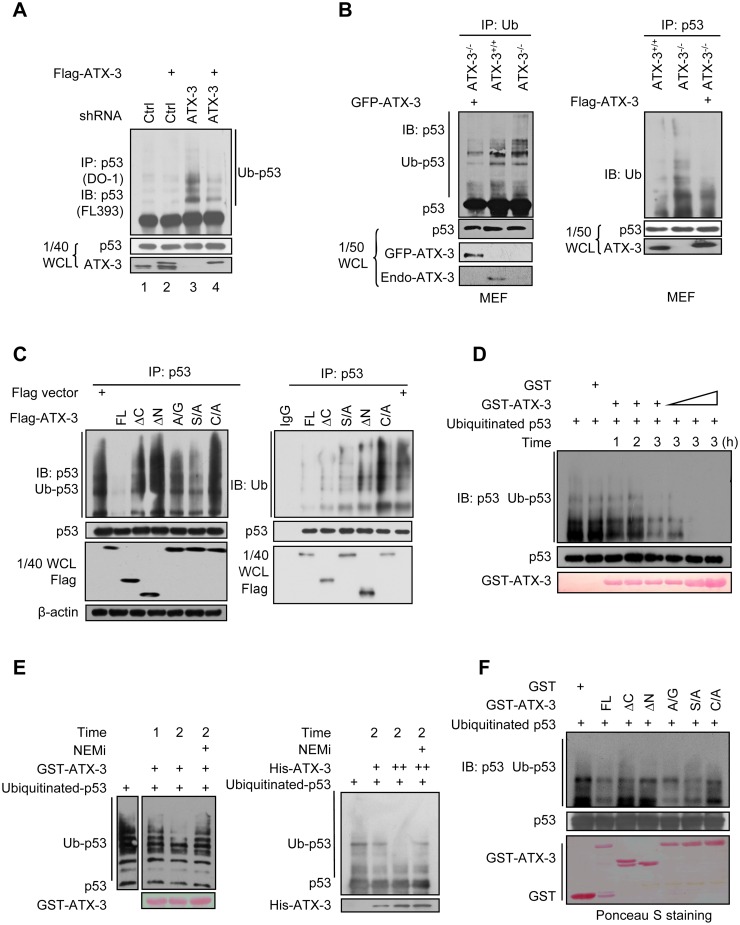
ATX-3 deubiquitinates p53. (A) HeLa cells stably expressing control shRNA and shRNA#1 were transiently transfected with control Flag empty vector or Flag-ATX-3. Twenty-four h later, cells were treated with MG132 (20 μM) for 4 h before harvest. Cell lysate was immunoprecipitated with anti-p53 (FL393) polyclonal antibodies and immunoblotted with monoclonal anti-p53 (DO-1) antibody. (B) ATX-3^+/+^ and ATX-3^-/-^ MEF cells as well as ATX-3^-/-^ cells transiently transfected with green fluorescent protein (GFP)-ATX-3 were treated with MG132 (20 μM) for 4 h before harvest. Cell lysate was immunoprecipitated either with anti-Ub polyclonal antibodies and immunoblotted with monoclonal anti-p53 (PAb421) antibodies (left panel) or with anti-p53 (FL393) polyclonal antibodies and immunoblotted with monoclonal anti-Ub antibody (right panel). Using both anti-Ub and anti-p53 antibodies confirmed the upper bands are ubiquitinated species of p53. (C) 293T cells were transfected with indicated constructs. Twenty-four h later, cells were treated with MG132 (20 μM) for 4 h before harvest. p53 was immunoprecipitated with anti-p53 (FL393) polyclonal antibodies and either immunoblotted with monoclonal anti-p53 (DO-1) antibodies (left panel) or immunoblotted with monoclonal anti-Ub antibody. (D) Ubiquitinated p53 was prepared as mentioned in Materials and Methods. GST and increasing amounts of GST-ATX-3 were incubated with ubiquitinated p53 in DUB buffer for indicated time. GST proteins were stained by Ponceau S, and p53 was immunoblotted with monoclonal anti-p53 (DO-1) antibodies. (E) Ubiquitinated p53 was incubated with indicated dose of purified ATX-3 in DUB buffer for indicated times. In one reaction tube, 10 mM of DUB inhibitor N-ethylmaleimide (NEMi) was added. GST and GST-ATX-3 were added and incubated with ubiquitinated p53 for indicated time in DUB buffer (left), and His-ATX-3 was added with ubiquitinated p53 and incubated for 2 h in DUB buffer (right). GST-fusion proteins were stained by Ponceau S, His-ATX-3 was immunoblotted with anti-His, and p53 was immunoblotted with monoclonal anti-p53 (DO-1) antibodies. (F) GST, GST-ATX-3 (FL), and the mutants were incubated with ubiquitinated p53 in DUB buffer. p53 was immunoblotted with monoclonal anti-p53 (DO-1) antibodies. GST proteins were stained by Ponceau S.

The interaction between ATX-3 and p53 suggested that p53 might be a substrate of ATX-3. Therefore, we tested whether ATX-3 affected the levels of p53 ubiquitination in vivo. As the p53 levels may differ among different cell lines, to show the generality of the effect of ATX-3 on p53, we generated ATX-3 stably knockdown cell lines in HeLa, HCT116, and RKO cells using two non-overlapping short hairpin RNA (shRNA) constructs. For the following experiments using knockdown cells in the study, either two clones of ATX-3 shRNA stably knockdown cells were used or one clone of knockdown cells was used but with rescue experiments performed in parallel. As indicated in Fig 2A, knockdown of ATX-3 in HeLa cells significantly increased the level of ubiquitinated-p53 (lane 3), and transient expression of ATX-3 effectively eliminated the increased ubiquitination that resulted from ATX-3 knockdown (lane 4). Moreover, ATX-3 overexpression resulted in a suppression of p53 ubiquitination (lane 2). Similar results were observed in ATX-3+/+ and ATX-3-/- mouse embryonic fibroblast (MEF) cells (Fig 2B left and right panel). Furthermore, our results showed that ATX-3 affected p53 ubiquitination in cells, and both the N-terminal Josephin domain and the C-terminal UIM domains of ATX-3 are required for this activity (Fig 2C). These results suggest that ATX-3 may act as a p53-directed DUB.

To determine whether ATX-3 can deubiquitinate p53 directly in vitro, we performed in vitro deubiquitination assays. Our results showed that FL ATX-3 deubiquitinated the ubiquitinated-p53 directly in a time- and dose-dependent manner (Fig 2D), which could be repressed by a nonspecific deubiquitinating inhibitor N-ethylmaleimide (NEMi) (Fig 2E left and right panel), and the effective deubiquitination of p53 required both the DUB activity and the poly-Ub binding ability of ATX-3 (Fig 2F).

### ATX-3 Stabilizes p53 via the Ub-Proteasome Pathway

**Fig 3:**
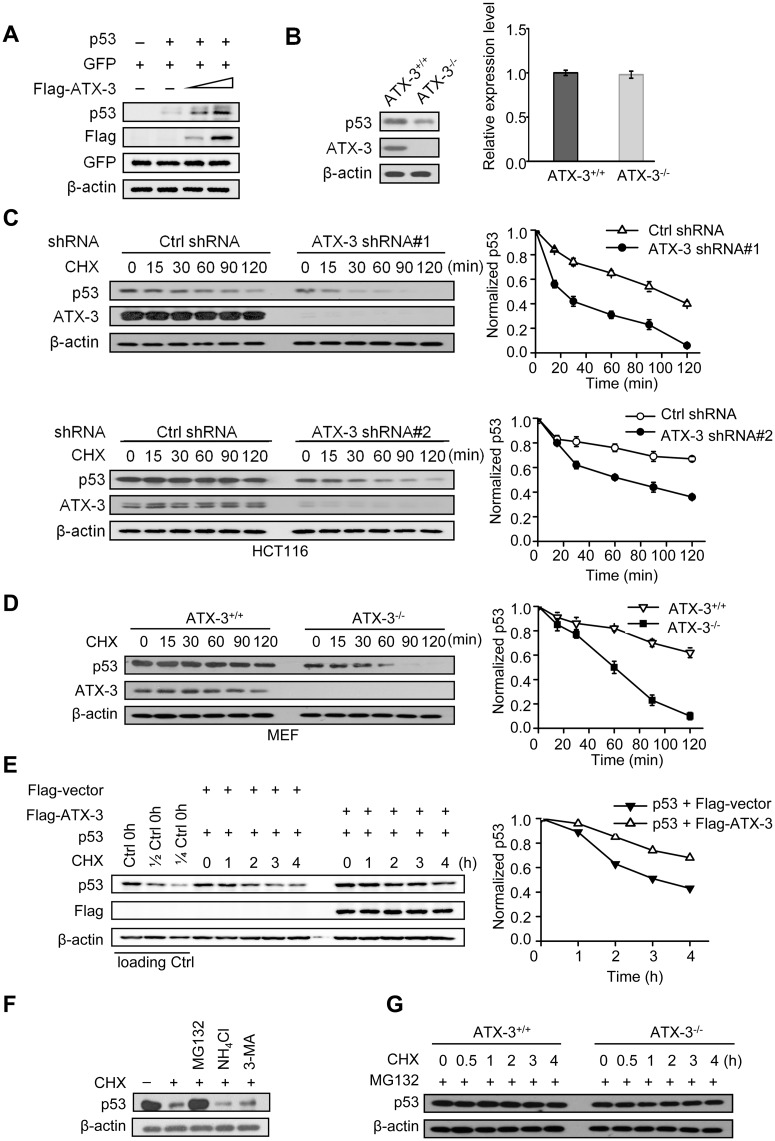
ATX-3 stabilizes p53 via the ubiquitin-proteasome pathway. (A) HCT116 p53^-/-^ cells were transfected with equal amounts of p53, GFP plasmids, and increasing amounts of Flag-ATX-3 plasmids. Cell lysates were subjected to immunoblotting with indicated antibodies. Levels of GFP were shown as equal transfection efficiencies, and β-actin was shown as loading control. (B) Lysates from ATX-3^+/+^ and ATX-3^-/-^ MEF cells were subjected to immunoblotting (left panel). Total RNA from ATX-3^+/+^ and ATX-3^-/-^ MEF cells was extracted and analyzed by quantitative reverse transcription-polymerase chain reaction (qRT-PCR) (right panel). The data represent the mean ± SD for three individual experiments. No statistical significance was observed between these two groups. Underlying data are shown in S1 Data. (C and D) Lysates from HCT116 cells stably expressing control shRNA or ATX-3 shRNA (shRNA #1, C-upper panel; shRNA #2, C-lower panel), and ATX-3^+/+^ and ATX-3^-/-^ MEF cells (D) treated with 20 μg/ml CHX for the indicated times were subjected to immunoblotting (left panels). p53 protein levels were quantified and normalized to β-actin. The data represent the mean ± SEM for three individual experiments (right panels). Underlying data are shown in S1 Data. (E) HCT116 p53^-/-^ cells were transfected with equal amounts of p53, Flag-ATX-3, and GFP plasmids. Twenty-four h later, cells were treated with 20 μg/ml CHX for the indicated times and subjected to immunoblotting (left panel). p53 protein levels were quantified and normalized to β-actin. The data are representative of one of the three independent experiments (right panel). Underlying data are shown in S1 Data. (F) Cells were left untreated or treated with CHX (20 μg/ml) for 30 min either alone or in the presence of the proteasomal inhibitor MG132 (20 μM) or the lysozyme inhibitor NH_4_Cl (20 mM) or the autophagy inhibitor 3-MA (5 mM). Cells were harvested and analyzed by immunoblotting. (G) Lysates from ATX-3^+/+^ and ATX-3^-/-^ MEF cells treated with 20 μg/ml of CHX together with 20 μM of MG132 for the indicated times were subjected to immunoblotting.

As ATX-3 interacts with p53 under physiological conditions and regulates the ubiquitination of p53 in cells, it is possible that ATX-3 may regulate the turnover of p53 via the Ub-proteasome pathway. We found that co-transfection of ATX-3 and p53 led to the accumulation of p53 protein compared with p53 alone-transfected cells (Fig 3A). In contrast, deletion of ATX-3 resulted in an overt reduction in p53 protein level (Fig 3B, left), with no appreciable change at the p53 mRNA level (Fig 3B, right), indicating that the regulation of p53 by ATX-3 is unlikely at the transcriptional level. p53 levels were also compared in ATX-3 wild-type (WT) and KO mice primary cultures by western blot (S1E Fig). The basal p53 levels in ATX-3 KO primary MEFs were significantly lower than those in WT primary MEF cells. ATX-3 regulates p53 posttranslationally, because the half-life of p53 was significantly shortened in the ATX-3 stably knocked-down HCT116 cells (Fig 3C upper). This result was further validated in another clone of ATX-3 shRNA-stable knockdown cell line (Fig 3C lower) as well as in ATX-3-/- MEF cells (Fig 3D). In HCT116 p53-/- cells, the ectopically expressed p53 showed significantly prolonged half-life in ATX-3 and p53 co-transfected cells compared to that in p53 alone-transfected cells (Fig 3E), thus confirming the positive regulatory effect of ATX-3 on p53 stability. Furthermore, we found that MG132, a proteasome inhibitor, but not NH_4_Cl, a lysosome inhibitor, or 3-methyladenine (3-MA), a well-characterized inhibitor of autophagy, could suppress p53 degradation after cycloheximide (CHX) treatment (Fig 3F). Pretreatment of cells with MG132 blocked the degradation of p53 in both ATX-3+/+ and ATX-3-/- MEF cells (Fig 3G). Taken together, these results indicate that ATX-3 stabilizes p53 in cells via the Ub-proteasome pathway.

### ATX-3 Regulates p53-Mediated Transcription and Cell Cycle Arrest

**Fig 4:**
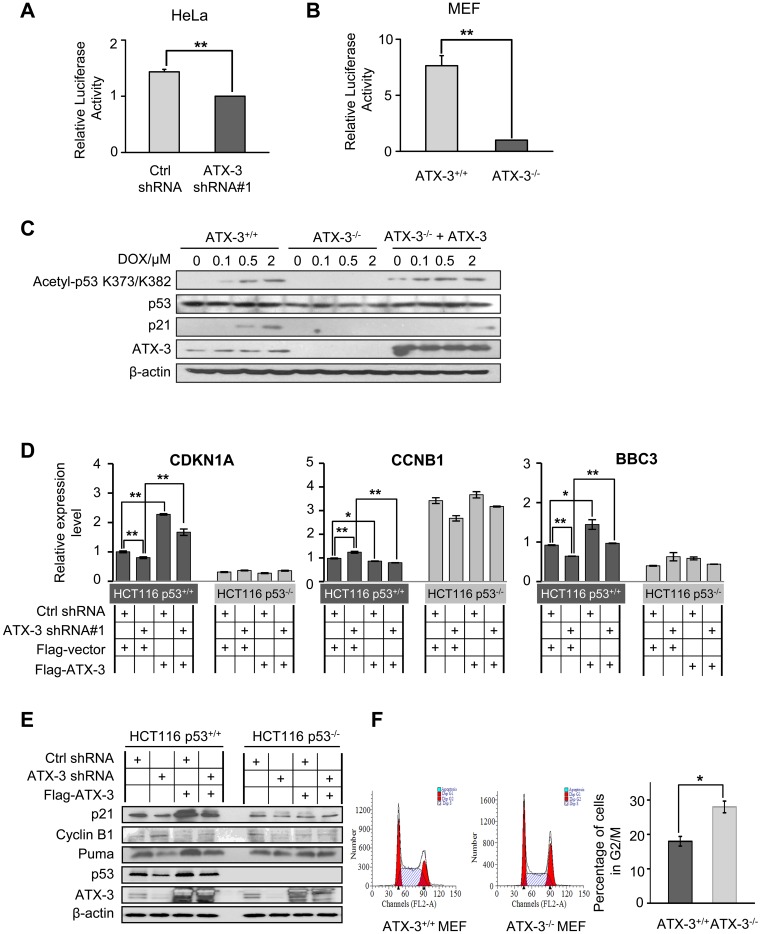
ATX-3 regulates p53-mediated transcription and cell cycle arrest. (A and B) PG13-luciferase reporter assay for p53 transactivation in ATX-3 stably knocked down HeLa cells (A) and ATX-3^+/+^ and ATX-3^-/-^ MEF cells. (B) Data were presented as mean ± SEM; *n* = 5 for HeLa cells and *n* = 8 for MEF cells. ** denotes *p* < 0.01. Underlying data are shown in S1 Data. (C) ATX-3^+/+^ and ATX-3^-/-^ MEF cells as well as ATX-3^-/-^ cells transiently transfected with Flag-ATX-3 were treated with different doses of DOX for 24 h. Cells were lysed, and total cell lysates were analyzed by western blotting with the indicated antibodies. Both acetylated p53 and p21 were used to monitor the activation of p53. (D and E) qRT-PCR (D) and western blot (E) analysis of p53 downstream targets in HCT116 p53^+/+^ and HCT116 p53^-/-^ control and ATX-3 stably knockdown cells, transfected with empty vector or plasmid encoding Flag-ATX-3. Relative mRNA levels were normalized to glyceraldehyde-3-phosphate dehydrogenase (GAPDH) (mean ± SEM; *n* = 3~6). * denotes *p* < 0.05, and ** denotes *p* < 0.01. Underlying data are shown in S1 Data. (F) ATX-3 deletion led to G2/M arrest. ATX-3^+/+^ and ATX-3^-/-^ MEF cells were fixed, stained with PI, and analyzed by flow cytometry. The data represent the mean ± SEM for six individual experiments. * denotes *p* < 0.05. Underlying data are shown in S1 Data.

To explore the functional consequences of ATX-3-modulated p53 stability, the normal functions of ATX-3 in regulating p53-dependent biological activities were tested. In dual luciferase reporter assay, the DNA binding ability of p53, which is measured by the fluorescence intensity of PG13-Luc reporter, was significantly decreased in both ATX-3 knockdown HeLa cells (Fig 4A) and ATX-3 KO MEF cell lines (Fig 4B). Furthermore, acetylation of p53 at K373/K382 is reported to be a marker for the stimulation of the p53 transactivation activity. We treated the MEF cells with doxycycline (DOX) and found that the levels of acetylated p53 and one of its target gene products, the cyclin-dependent kinase inhibitor p21^cip1/waf^ proteins, were remarkably decreased in ATX-3^-/-^ MEF cells compared to ATX-3^+/+^ controls, and this could be restored to normal levels by transient expression of ATX-3 (Fig 4C). These results suggest that ATX-3 deletion inhibits the stimulation of p53 transactivation activity. The expression of several p53 target genes, for example, *CDNK1A*, *CCNB1*, and *BBC3*, was affected at both mRNA (Fig 4D and S2A Fig) and protein levels (Fig 4E and S2B Fig) associating with ATX-3 levels in HCT116 and MEF cells. The mRNA levels of cyclin B1 were higher in ATX-3 knockdown cells than that in control cells. This is because p53 acts as a repressor for cyclin B1. ATX-3 knockdown leads to an inhibition of p53 transcriptional activities, thus relieving its suppression on cyclin B1, resulting in its increased expression. Notably, overexpression of ATX-3 induced up-regulations of *CDNK1A* and *BBC3* but a down-regulation of *CCNB1*, which required both the DUB activity and the poly-Ub binding ability of ATX-3 (S2C–S2F Fig). In contrast, knockdown of ATX-3 significantly blocked their induction, and this could be restored upon ATX-3 ectopic expression (Fig 4D and 4E). The inductions of all three p53 target genes were not changed in HCT116 p53^-/-^ cells, indicating that these inductions were p53 dependent (Fig 4D and 4E).

In addition, fluorescence-activated cell sorting (FACS) analysis of cell cycle showed that ATX-3 deletion resulted in an increased proportion of cells in G2/M phase (Fig 4F). Without p53 and ATX-3 double KO MEF cells in hand, we examined the p53-dependence of this effect by using ATX-3 stably knocked down HCT116 p53^+/+^ and HCT116 p53^-/-^ cells. An increase of G2/M phase cells was observed in ATX-3 knockdown HCT116 p53^+/+^ cells but not in HCT116 p53^-/-^ cells, suggesting that ATX-3 was involved in the regulation of cell cycle arrest in G2/M phase, which was also p53 dependent (S2G Fig). Therefore, our data demonstrated that ATX-3 was able to regulate p53-dependent gene expressions and cell cycle arrest.

### ATX-3 Promotes p53-Dependent Apoptosis in Cells and in Zebrafish

**Fig 5:**
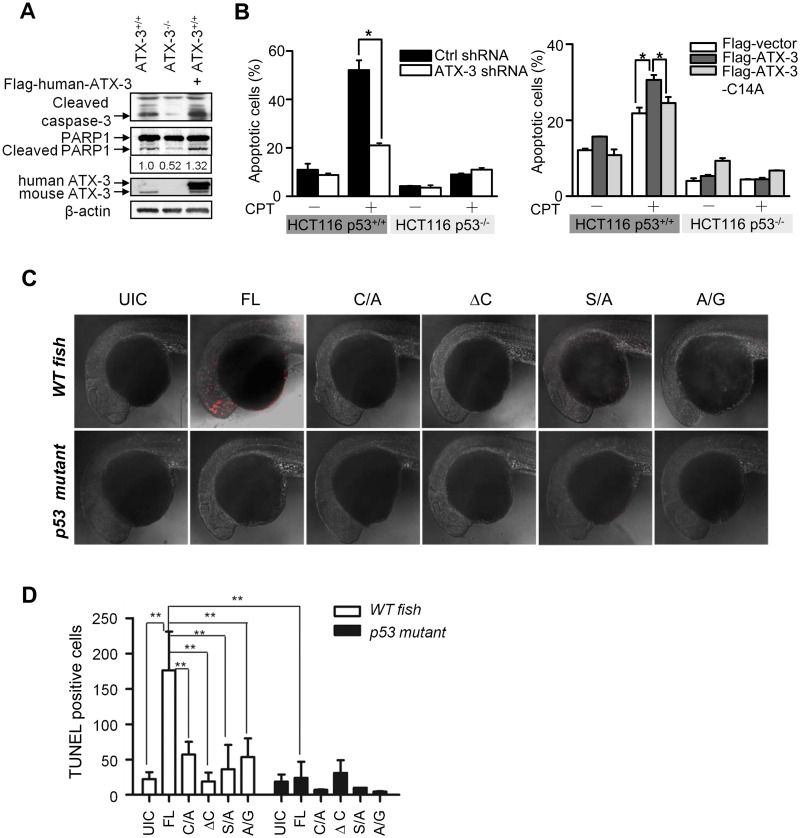
ATX-3 promotes p53-dependent apoptosis in cells and in zebrafish. (A) Western blot analysis of the involvement of ATX-3 in apoptosis as measured by the detection of caspase-3 and PARP1 cleavage in ATX-3^+/+^ and ATX-3^-/-^ MEF cells, transfected with empty vector or plasmid encoding Flag-ATX-3. The relative changing fold of the cleaved PARP1 was underlined. (B) HCT116 p53^+/+^ and HCT116 p53^-/-^ cells, stably shRNA-expressing cells, or cells transiently transfected with Flag-vector or Flag-ATX-3 or Flag-ATX-3-C14A were treated with or without CPT (1 μM) for 24 h. Cells were analyzed by flow cytometry for apoptosis using Annexin V/PI. Results are the mean ± SEM of three independent experiments. * denotes *p* < 0.05. Underlying data are shown in S1 Data. (C) *WT* and *p53 mutant* zebrafish embryos were injected with indicated object mRNAs (100 pg) at the one-cell stage and harvested at 24 h post fertilization (hpf) for TUNEL labeling and observed by confocal microscopy. Embryos were shown in lateral view with anterior to the left. UIC, uninjected control. (D) Quantitative analysis of Fig 5C. The data represent the mean ± SEM for TUNEL-positive cells in 3~9 zebrafish embryos. ** denotes *p* < 0.01. Underlying data are shown in S1 Data.

As p53 is a well-established apoptosis-regulator, we next examined whether ATX-3 affected p53-dependent apoptosis. We found that hallmarks of apoptosis, including the cleaved caspase-3 and poly (ADP-ribose) polymerase (PARP1), were less in ATX-3^-/-^ MEF cells, while overexpression of ATX-3 resulted in significant caspase-3 and PARP1 cleavage (Fig 5A), indicating that ATX-3 was involved in the regulation of apoptosis in cells. Flow cytometry analysis using Annexin V-FITC/propidium iodide (PI) staining in HCT116 cells showed that knockdown of ATX-3 led to a decrease of camptothecin (CPT)-induced apoptosis, while ectopic expression of ATX-3 but not the catalytic inactive mutant ATX-3-C14A resulted in a significant increase of apoptosis in HCT116 p53^+/+^ but not HCT116 p53^-/-^ cells (Fig 5B), indicating that ATX-3 promoted p53-mediated apoptosis, which required its deubiquitinating enzymatic activity.

Using the zebrafish model system, we further examined whether ATX-3 induces p53-dependent apoptosis in vivo. As our cellular results showed that knockdown of ATX-3 led to a significant decrease of CPT-induced apoptosis, it is quite possible no apoptosis signal would be detected under unperturbed conditions when ATX-3 is knocked down or knocked out. Therefore, the apoptosis as well as neurodegeneration in zebrafish were performed under ectopic expression conditions instead of knockdown or KO conditions. *p53 WT* and *mutant* zebrafish embryos were injected with mRNA of FL and various *ATX-3* mutants. Twenty-four h post injections, the embryos were harvested and TUNEL-positive cells were analyzed. When the FL *ATX-3* mRNA was injected into *WT* but not the *p53 mutant* zebrafish embryos, significantly more apoptotic cells were observed in TUNEL-staining assays, indicating that ATX-3 caused p53-dependent apoptosis in vivo. In addition, injections of mRNA of the catalytic inactive mutant *ATX-3-C14A (C/A)*, the C-terminal deletion (*ΔC*), and the two UIM mutants (*S/A* and *A/G*) *ATX-3* in *WT p53* zebrafish embryos exhibited significant reduction in apoptosis compared with that of FL *ATX-3*, and no apoptosis was observed in *p53 mutant* zebrafish, indicating that the induction of p53-dependent apoptosis was critically dependent on the catalytic activity and the UIM domain of ATX-3 (Fig 5C and 5D). Interestingly, we observed that most TUNEL-positive cells localized in the head area of the zebrafish, suggesting that the apoptosis may occur mainly in the nervous system. To confirm this, TUNEL assay was performed by using the *Tg(HuC*:*EGFP)* transgenic zebrafish embryos, in which GFP-positive cells represent the expression of zebrafish neuronal marker *elavl3* (formerly known as *HuC*) [33]. We observed that the TUNEL-positive cells induced by the FL *ATX-3* mRNA injection were localized in GFP-positive brain regions (telencephalon, S3B Fig; diencephalon/hindbrain, S3C Fig) of the zebrafish, indicating that the *ATX-3* mRNA injection-induced apoptosis occurred mainly in the nervous system of zebrafish.

### The Interaction and DUB Activity of ATX-3 to p53 Are Functionally Enhanced by PolyQ Expansion

**Fig 6:**
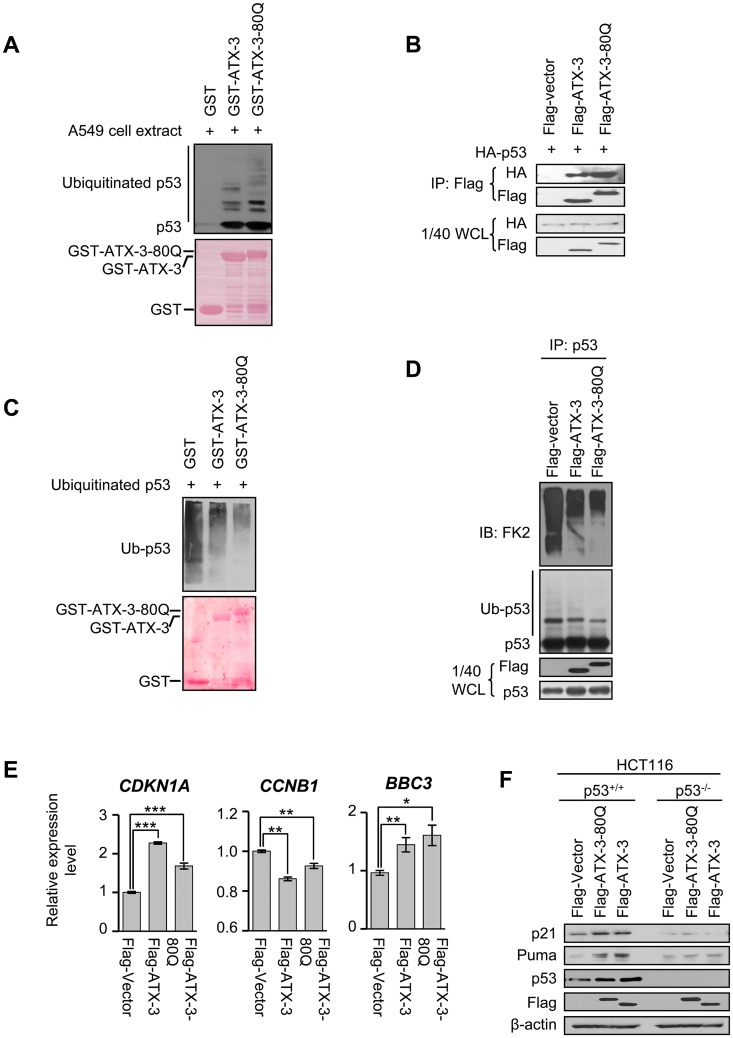
The interaction and DUB activity of ATX-3 to p53 are functionally enhanced by polyQ expansion. (A) GST—pull-down assay was performed with the indicated GST-fusion proteins and the whole cell extract of A549. GST proteins were stained by Ponceau S, and the pulled down proteins were analyzed by immunoblotting with anti-p53 polyclonal antibody. (B) 293T cells were collected 24 h after they were co-transfected with HA-p53 and Flag-vector or normal Flag-ATX-3 or Flag-ATX-3-80Q. Cell lysate was immunoprecipitated with anti-Flag M2 affinity gel and immunoblotted with polyclonal anti-HA and anti-Flag antibody. (C) GST, GST-ATX-3, and GST-ATX-3-80Q were incubated with ubiquitinated p53 in DUB buffer for 2 h at 37°C. GST proteins were stained by Ponceau S, and p53 was immunoblotted with monoclonal anti-p53 (DO-1) antibodies. (D) 293T cells were transiently transfected with control Flag vector or normal Flag-ATX-3 or Flag-ATX-3-80Q. Twenty-four h later, cells were treated with MG132 (20 μM) for 4 h before harvest. Cell lysate was immunoprecipitated with anti-p53 polyclonal antibodies (FL393) and immunoblotted with monoclonal anti-Ub (FK2) and anti-p53 (DO-1) antibody. (E and F) qRT-PCR (E) and western blot (F) analysis of p53 downstream targets in HCT116 p53^+/+^ and HCT116 p53^-/-^ control and ATX-3 stably knocked down cells, transiently transfected with empty vector or plasmid encoding normal Flag-ATX-3 or Flag-ATX-3-80Q. Relative mRNA levels were normalized to GAPDH (mean ± SEM; *n* = 3~7). * denotes *p* < 0.05, ** denotes *p* < 0.01, *** denotes *p* < 0.001. Underlying data are shown in S1 Data.

PolyQ-expanded ATX-3 (ATX-3^exp^ (80Q)) is thought to undergo conformational changes and acquire toxic properties, leading to altered molecular interactions. We wonder whether the polyQ expansion affects the ATX-3/p53 interaction. GST—pull-down assay (Fig 6A) and coimmunoprecipitation experiments (Fig 6B) showed that p53, both native and ubiquitinated form, bound ATX-3^exp^ (80Q) stronger than the normal ATX-3. Consistently, ATX-3^exp^ (80Q) exhibited stronger DUB activity than the normal ATX-3 in vitro (Fig 6C) and in cells (Fig 6D). Besides, we found that the degradation of p53 in the ATX-3^exp^ (80Q)-expressing cells was slower than that of normal ATX-3-expressing cells (S4C and S4D Fig), and ectopic expression of polyQ-expanded ATX-3 induced higher levels of p53 protein than the normal ATX-3 in RKO, 293T, and MEF cells (S4E Fig), indicating that polyQ-expanded ATX-3 possessed enhanced capability to stabilize p53. The expression levels of p53-responsive genes (such as p21 and Puma) were also higher in ATX-3^exp^ (80Q) expressing RKO cells (S4F Fig), suggesting that p53 was functionally enhanced by polyQ expansion in ATX-3. For unknown reasons, HCT116 cells do not behave as significantly as other cell lines (such as RKO, 293T, and MEFs) in terms of p53 induction (Fig 6F and S4E Fig). To determine the p53 dependence issue, we used the HCT116 cell lines with both p53^+/+^ and p53^-/-^ genotypes. In HCT116 p53^+/+^, but not HCT116 p53^-/-^ cells, both the normal and polyQ-expanded ATX-3 increased the induction of p53-responsive genes at both mRNA (Fig 6E and S4A Fig) and protein levels (Fig 6F and S4B Fig) when compared to the empty vector control group, but the difference did not reach the statistical significance between the normal ATX-3 group and polyQ-expanded ATX-3 group. All together, these data indicated that the polyQ expansion did not disturb the binding and the DUB activity of the normal ATX-3 to p53 and appeared to augment p53 stabilization.

### PolyQ Expansion Affects p53-Dependent Apoptotic Function in Cells and in Zebrafish

**Fig 7:**
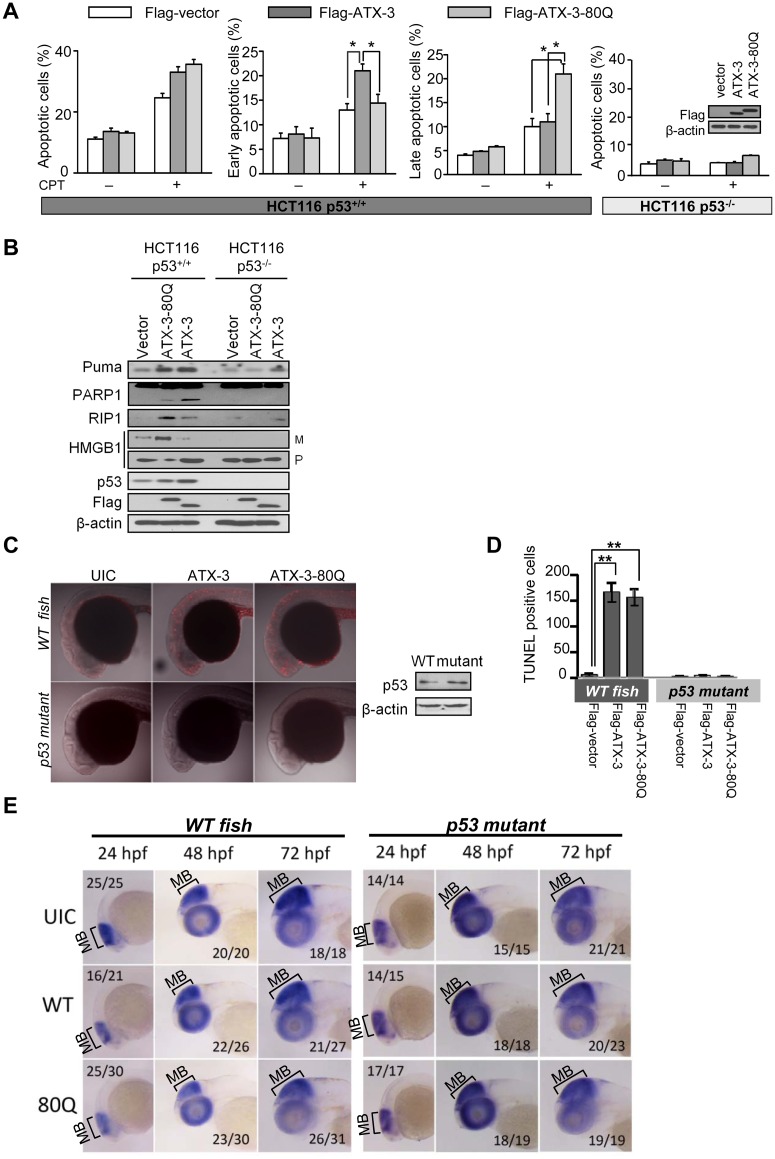
PolyQ expansion affects p53-dependent apoptotic function in cells and in zebrafish. (A) HCT116 p53^+/+^ and HCT116 p53^-/-^ cells transiently transfected with Flag-vector or normal Flag-ATX-3 or Flag-ATX-3-80Q were treated with CPT (1 μM) for 24 h and then analyzed by flow cytometry for apoptosis using Annexin-V/PI. Results are the mean ± SEM of three independent experiments. * denotes *p* < 0.05. Cell lysates were immunoblotted with indicated antibodies. Underlying data are shown in S1 Data. (B) HCT116 p53^+/+^ and HCT116 p53^-/-^ cells transiently transfected with Flag-vector or the normal Flag-ATX-3 or Flag-ATX-3-80Q were treated with CPT (1 μM) for 24 h. Lysates and proteins in the medium were analyzed by immunoblotting with indicated antibodies. M, released into medium; P, pellet. (C and D) Flag-ATX-3-80Q induced p53-dependent apoptosis in zebrafish. Zebrafish embryos were injected with 100 pg of *Flag-ATX-3 (ATX-3)* or *Flag-ATX-3-80Q (ATX-3-80Q)* mRNA at one-cell stage. UIC, uninjected control. Twenty-four hpf, embryos were subjected to TUNEL assay and observed by confocal microscopy. Endogenous p53 levels in the *p53 WT* and *p53 mutant* zebrafish were examined by western blot. (C) TUNEL-positive cells were quantified from 5~7 embryos each. Results are the mean ± SEM. ** denotes *p* < 0.01, compared with the control. (D). Underlying data are shown in S1 Data. (E) Whole-mount in situ hybridization analyses of the midbrain neural marker *otx2* in uninjected control (UIC) or *normal ATX-3 (WT)* or *ATX-3*^*exp*^ *(80Q)* mRNA-injected *p53 WT* (left) and *p53 mutant* (right) zebrafish embryos at 24, 48, and 72 hpf. Embryos were shown in lateral views with anterior to the left. The ratio of embryos with the representative phenotypes was indicated. Both *WT* and *80Q* mRNA injections resulted in obvious *otx2* signal decreases in *p53 WT* but not *p53 mutant* zebrafishes, with more profound *otx2* signal reduction in *80Q* mRNA injections. MB denotes midbrain.

CPT-induced apoptosis was analyzed by flow cytometry using Annexin V/PI. Apoptotic cells are those positive for Annexin V staining (either positive or negative for PI staining) (S3A Fig). Our results showed that expression of the FL ATX-3^exp^ (80Q) led to a similar increase of p53-dependent apoptosis (including early and late apoptosis/necrosis) in HCT116 p53^+/+^ (Fig 7A) and zebrafish (Fig 7C and 7D) comparing to the normal ATX-3. Early apoptotic cells are Annexin V+/PI- staining, whereas late apoptotic/necrotic cells are Annexin V+/PI+ staining (PI-positive staining is due to a loss of plasma membrane integrity). Interestingly, we found that the normal ATX-3 induced a significantly higher percentage of early apoptotic cells, whereas the ATX-3^exp^ (80Q) led to more late apoptotic/necrotic cells in HCT116 p53^+/+^ but not HCT116 p53^-/-^ cells (Fig 7A and S3A Fig). In support, we found that CPT treatment resulted in clearly different morphological nuclear changes in the ATX-3^exp^ (80Q)- and the normal ATX-3-expressing HCT116 p53^+/+^ but not HCT116 p53^-/-^ cells after staining the nuclear DNA by Hoechst-33342 (S5A Fig). Nuclei of the normal ATX-3-expressing cells were round shaped but without condensed and fragmented chromatin, which represent an early apoptotic event. In contrast, nuclei of ATX-3^exp^ (80Q)-expressing cells looked more amorphous, without any defined surface outline, and the nuclear heterochromatin was extensively packaged, indicating necrotic cell death (S5A Fig). Furthermore, as shown in Fig 7B, the p53-dependent apoptosis program marker such as cleaved PARP-1 in the ATX-3^exp^ (80Q)-expressing HCT116 p53^+/+^ cells was found to be higher than the empty vector control group but weaker when compared to the normal ATX-3 group, indicating that the mitochondrial apoptotic p53 program played a role in this process, but the extent of this influence may not be as profound as that of the normal ATX-3. More importantly, High Mobility Group Box 1 (HMGB1) protein that released into the culture medium (a classical biochemical hallmark specific for necrosis [34,35]) as well as the level of receptor-interacting serine/threonine protein kinases 1 (RIP1, an important mediator of necrosis) were found to be significantly enhanced in ATX-3^exp^ (80Q)-expressing HCT116 p53^+/+^ cells compared to those of the empty vector control and the normal ATX-3 group after CPT treatment, indicating that CPT apparently triggered a necrotic program in addition to the mitochondrion-dependent apoptotic program in polyQ-expanded ATX-3-expressing cells. These markers were not significantly changed in HCT116 p53^-/-^ cells (Fig 7B), demonstrating the changes of these markers were p53 dependent. Together, these results suggested that a continuum of apoptosis and necrosis existed in response to CPT insult in ATX-3^exp^ (80Q)-expressing cells, both of which were mediated by p53.

Next, we set out to determine if polyQ-expanded ATX-3 causes more neuronal cell death in vivo by using zebrafish as a model vertebrate. We injected mRNA of the normal *ATX-3* and *ATX-3*^*exp*^ (80Q) into *wild-type* and *p53 mutant* fish one-cell embryos. In zebrafish, *otx2* is expressed in the prospective forebrain/midbrain in mid/gastrulae [36–38], and *neurogenin 1* (*ngn1*) is reported to be a determinant of zebrafish basal forebrain dopaminergic neurons [39,40]. We performed the whole-mount in situ hybridization using *otx2* and *ngn1* as two neural markers to evaluate the neural loss upon the normal *ATX-3* and *ATX-3*^*exp*^*(80Q)* expression in zebrafish. We observed that expression of normal ATX-3 or ATX-3^exp^ (80Q) resulted in decreased signals of *otx2* (blue staining mainly in MB area, Fig 7E and S5B Fig) and *ngn1* (blue staining including telencephalon (TE), midbrain (MB), and hindbrain (HB) areas, S5C Fig) in *WT* but not *p53 mutant* zebrafishes at 24 h post fertilization (hpf), with more profound reduced signals of *otx2* and *ngn1* in *ATX-3*^*exp*^ *(80Q)* mRNA injection groups (Fig 7E, S5B and S5C Fig). By 48 and 72 hpf, brains of *ATX-3*^*exp*^ *(80Q)*-injected zebrafishes showed even weaker levels of both *otx2* and *ngn1* (Fig 7E, S5B and S5C Fig). These results provided clear evidences that expression of ATX-3^exp^ (80Q) led to more neuronal loss in brains of *WT* but not *p53 mutant* zebrafishes, suggesting that ATX-3^exp^ (80Q) caused more severe neuronal degeneration in SCA3 in a p53-dependent manner.

### PolyQ Expansion of ATX-3 Causes p53-Dependent Neurodegeneration in Mice Brains

**Fig 8:**
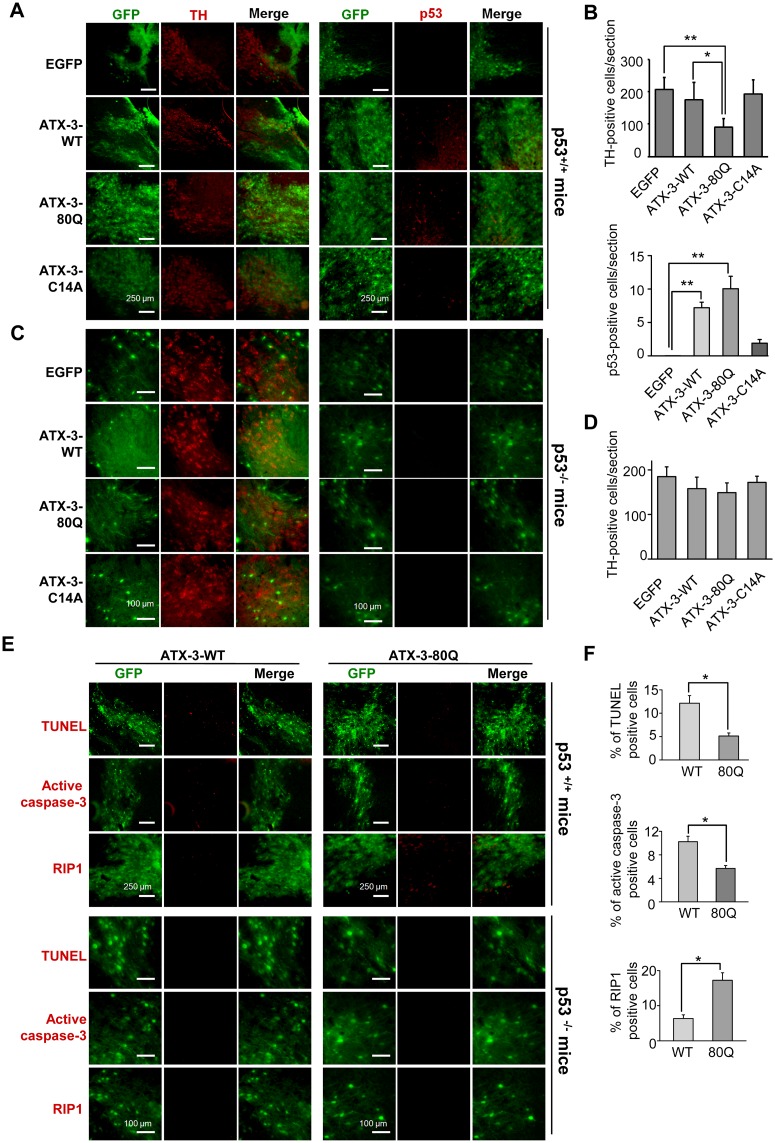
PolyQ expanded ATX-3 caused p53-dependent neurodegeneration in the SNpc of transgenic mice. (A and C) Immunostaining staining of TH (left panel) and p53 (right panel) in the SNpc of p53^+/+^ (A) and p53^-/-^ (C) mice. Scale bars were 250 μm for p53^+/+^ mice and 100 μm for p53^-/-^ mice. (B) Quantitative analysis of the number of TH-positive and p53-positive neurons in p53^+/+^ mouse brains injected in the SNpc with LV. Values are presented as means ± S.D. of 3 mice per group of 1–3 sections per mouse. * denotes *p* < 0.05 and ** denotes *p* < 0.01. Underlying data are shown in S1 Data. (D) Quantitative analysis of the number of TH-positive neurons in p53^-/-^ mouse brains injected in the SNpc with LV. Values are presented as means ± S.D. of three mice per group of one or two sections per mouse. No statistical significance was observed among these groups. Underlying data are shown in S1 Data. (E) Assessment of neuronal cell death in the SNpc of p53^+/+^ and p53^-/-^ mice. Brain sections through the SNpc were stained using the TUNEL method or anti-activated caspase-3 antibody or anti-RIP1 antibody (red panel). Images shown in this figure are representative of three repeated experiments. Scale bars were 250 μm for p53^+/+^ mice and 100 μm for p53^-/-^ mice. (F) Quantitation of the percentage of TUNEL-positive signals or the percentage of cells positive for activated caspase-3 or RIP1 in the population of EGFP-positive neurons in p53^+/+^ mice. Values are presented as means ± S.D. *n* = 3. * denotes *p* < 0.05. Underlying data are shown in S1 Data.

Previous studies have shown that neurodegeneration affects particular brain regions in MJD pathology [41]. To further study the neurodegeneration in specific affected brain regions in MJD, we generated an in vivo MJD genetic mouse model by ectopic expression of WT and mutant ATX-3 (80Q and C14A) with an enhanced green fluorescent protein (EGFP) tag in the substantia nigra pars compacta (SNpc) or striatum of p53^+/+^ and p53^-/-^ mouse brain using lentiviral vectors (LV). The expression of these LV was first validated in 293T cells using fluorescence microscopy (S6A Fig) and also by western blot with anti-ATX-3 antibody (S6B Fig). Immunostaining analysis for tyrosine hydroxylase (TH), a marker for dopaminergic neurons in the SNpc, and for dopamine- and cyclic AMP-regulated neuronal phosphoprotein (DARPP-32), a regulator of dopamine receptor signaling, was performed to evaluate the neurodegeneration induced by lentiviral transduction. Our results showed that, in p53^+/+^ mice, WT ATX-3 caused a nonsignificant reduction of 15% TH-positive neurons in the SNpc (Fig 8A and 8B) and a reduction of 31% DARPP-32–positive neurons in the striatum (S6C and S6D Fig), whereas mutant ATX-3^exp^ (80Q) induced a more significant loss of neurons in both of these two brain areas when compared to the empty vector expressed brain section, with a reduction of 57% for TH-positive neurons in the SNpc (Fig 8A and 8B) and of 51% for DARPP-32–positive neurons in the striatum (S6C and S6D Fig). The loss of TH or DARPP-32 immunointensity mainly occurred in EGFP-positive neurons. No significant neuronal loss was detected in either the SNpc or the striatum of p53^+/+^ mice injected with the catalytic inactive mutant ATX-3-C14A (Fig 8A and 8B, S6C and S6D Fig). The evidences for p53 involvement in the mutant ATX-3^exp^ (80Q)-induced neurodegeneration were provided by the fact that no significant decreases of both neuronal markers were detected in p53^-/-^ mice after LV transduction (Fig 8C and 8D, S6E and S6F Fig). Further supports for p53 involvement were evidenced by the observation that WT and mutant ATX-3^exp^ (80Q), but not the catalytic inactive mutant C14A, caused marked increases in p53 immunoreactivity in the SNpc (Fig 8A and 8B) and striatum (S6C and S6D Fig) of p53^+/+^ mice, whereas no p53 staining was detected in the p53^-/-^ mice (Fig 8C and 8D, S6E and S6F Fig). These results provided consistent evidences for a role of p53 in the WT and mutant ATX-3^exp^ (80Q) expression induced neuronal degeneration.

To determine whether loss of TH staining in the SNpc as well as DARPP-32 staining in the striatum, associated with increased expression of WT and mutant ATX-3^exp^ (80Q), was due to neuronal death, we performed TUNEL analysis and monitored expression of activated caspase-3. The numbers of TUNEL-positive and activated caspase-3-positive cells were significantly increased in the EGFP-positive neurons in both the SNpc (Fig 8E and 8F) and striatum (S7A and S7B Fig) of p53^+/+^ but not p53^-/-^ mice. These results confirmed the association of cell death with WT and mutant ATX-3^exp^ (80Q) expression. It should be noted that WT ATX-3 induced significant higher TUNEL and activated caspase-3 staining compared to the mutant ATX-3^exp^ (80Q). As signs of non-apoptotic cell death, including extracellular release of HMGB1 and higher expression of RIP1, were observed in mutant ATX-3^exp^ (80Q)-expressed cells (Fig 7B), and it is generally accepted that activated caspase-3 are rarely detected in the case of necrosis [42], we hypothesized that necrosis might occur in these brain areas. To gain further insights into mechanism of the neuronal death, we evaluated the expression of RIP1, which is an important molecule mediating necrosis when caspases are inhibited [43]. We found that mutant ATX-3^exp^ (80Q)-expressed brain sections of p53^+/+^ but not p53^-/-^ mice had more intense staining for RIP1 than did the WT ATX-3 sections (Fig 8E and 8F, S7A–S7C Fig). A high magnification view of the anti-RIP1 and anti-TH immunostaining in the SNpc of p53^+/+^ mice showed that most TH-positive neurons showed intact nuclear membrane, whereas those neurons that had condensed chromatin structures and nuclei were highly RIP1 positive but negative for TH staining (S7C Fig). No obvious RIP1 immunostaining was observed in p53^-/-^ mice (S7C Fig). These results indicated the involvement of RIP1 in polyQ ATX-3^exp^ (80Q)-induced neuronal death in mouse brains. Together, our in vivo data have demonstrated that the polyQ ATX-3 caused p53-dependent neuronal death in both apoptotic and necrosis manner in mouse brains.

## Discussion

p53 activity is crucial in determining the cellular fate, keeping a delicate balance between cancer-suppressive and age-promoting functions [44–48]. Therefore, tight regulation of p53 is essential for maintaining normal cellular functions. It has been shown that p53 is mainly regulated at the level of protein stability, which occurs predominantly through the Ub-mediated proteasomal degradation. On the flip side of the regulation, deubiquitination, which is mediated by DUBs, provides a parallel important regulatory control of p53 stability. Previously, several DUBs from the ubiquitin-specific protease (USP) [49–53] and otubain (OTU) family members [54] have been shown to regulate the Mdm2-p53 pathway, each with different detailed mechanisms of action. For example, USP7 (also named HAUSP) was the first identified USP that stabilizes p53 [55]. Later, it was found to deubiquitinate Mdm2 and Mdmx as well [56], thus showing selective deubiquitination to regulate the homeostatic levels of p53, Mdm2, and Mdmx under both normal and stress conditions [55]. Unlike USP7, USP10 [52], a cytoplasmic DUB, had recently been shown to directly deubiquitinate p53, but not Mdm2 and Mdmx, and to regulate the subcellular localization and stability of p53 by opposing the effects of Mdm2. In the present study, we report that p53 is a novel substrate of ATX-3 under physiological conditions.

Previous studies have suggested a possible role of the tumor suppressor protein p53 in neurodegenerative diseases, although the evidences are indirect. p53 is mutated in approximately half of all human cancers, and accumulating evidences also supported a role of p53 in neurodegeneration [57,58]. For example, p53 was found to be highly elevated in brains affected by several neurodegenerative diseases, including Alzheimer’s disease (AD), Parkinson’s disease (PD), Huntington’s disease (HD), amyotrophic lateral sclerosis (ALS), HIV-associated neurocognitive disorders (HAND), etc. [59]. Furthermore, several epidemiological studies have found an inverse correlation between the risk of developing neurodegenerative disorders and cancer [60–62], suggesting that some common protein effectors might likely be involved between these two multifactorial chronic pathologies. Given the important role of p53 in neurodegenerative diseases and cancer, it is thus a likely possible candidate. Recent studies have reported that ATX-3 and ATX-3 like are involved in gastric cancer [63] and breast cancer [64], supporting the association of the Josephin family of DUBs with cancer. Importantly, aberrant activation of the p53 pathway has previously been reported in both MJD patient brain tissues and transgenic animal disease models [65–69], and elevated p53 level was observed in MJD transgenic mice [65]. Therefore, the overall above-cited data concur to suggest the possibility of a functional link between ATX-3 and p53. p53 has not come out as a potential issue in the ATX-3 KO mice from the literature. As we mentioned in the Introduction section, this may be because other members of the MJD DUBs may compensate for its absence in ATX-3 KO models under unstressed conditions. Here in our study, we discovered that ATX-3 interacts with p53 and functions as a DUB for p53.

**Fig 9:**
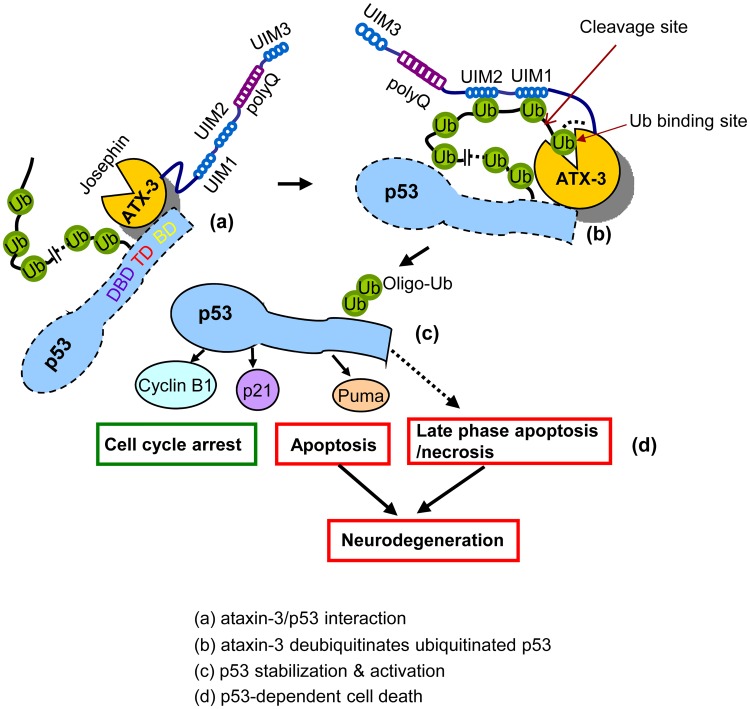
A schematic diagram for ATX-3 interaction and regulation of p53. (A) ATX-3 binds to the DNA-binding domain and the C-terminal regulatory domain of p53 via its Josephin domain under physiological conditions. (B) During the DUB process, ATX-3 primarily binds to ubiquitinated p53 through UIM1 and UIM2, and the Ub binding sites in the Josephin domain may also help the binding. (C and D) The deubiquitination of p53 by ATX-3 in physiological conditions leads to the stabilization of p53, which further results in the selective activation of p53-responsive genes involving both cell cycle arrest and apoptosis. The expansion of the C-terminal polyQ tract augments the binding of ATX-3^exp^ (80Q) with p53 and thus enhances the DUB activity of ATX-3^exp^ (80Q), resulting in p53-dependent cell death (including both early apoptotic and late apoptotic/necrotic manner) in SCA3 neurons.

We have observed that the Josephin domain of ATX-3 is sufficient for the direct binding of ATX-3 to native p53, whereas the ubiquitinated p53 interacts with ATX-3 primarily through the UIMs, indicating that UIMs function to help recruit and bind the ubiquitinated p53 (Fig 9A and 9B). Therefore, both the Josephin and UIM domain coordinately regulate the interaction between ATX-3 and p53 (Fig 9A and 9B). During the DUB process, in addition to the catalytic cysteine 14 site and the N-terminal Josephin domain, the UIMs of ATX-3 are also required for its DUB activity towards p53, which may function to position the polyubiquitinated p53 correctly relative to the catalytic site for subsequent cleavage (Fig 9B). Thus, ATX-3 deubiquitinates and stabilizes p53 (Fig 9C), which is an essential step for p53 function in cell cycle arrest and apoptosis (Fig 9D). The direct interaction between ATX-3 and p53 is primarily mediated by the Josephin domain, and the first two UIM domains function to enhance the interaction by trapping the Ub-chains on p53. The polyQ tract between UIM2 and UIM3 is expanded in MJD. Our results suggest that polyQ length enhances rather than disturbs the binding and deubiquitination of ATX-3^exp^ to p53 (Fig 6A–6D), which, in turn, causes more p53-dependent apoptosis/necrosis (Figs 7A, 7B, 7E, 8E and 8F, S5B, S5C, S7A and S7B Figs).

Previously, mutant huntingtin with expanded polyQ was found to bind to p53 and cause more cell death than WT huntingtin in neuronal cultures [70]. Here, polyQ-expanded ATX-3 was found to cause an increased percentage of cells undergoing p53-dependent late apoptotic/necrotic cell death than the normal ATX-3 did in HCT116 cells (Fig 7A and 7B, S3A Fig) and in neurons (Fig 8E and 8F). In consistence with our result, Evert’s group also observed an increased necrotic cell death in a cellular model for SCA3 upon polyQ ATX-3 expression [71], and p53 have been recently reported to play important roles in activating necrotic cell death [35,72,73]. Whether polyQ-expanded ATX-3 has additional targets that work together with p53 in inducing necrosis is not known yet, but we did find that the level of RIP1, an important mediator in necrosis, increased significantly in p53^+/+^ cells upon polyQ-expanded ATX-3 expression when compared to the empty vector control and the normal ATX-3 group, but remained at a basal low level in p53^-/-^ cells (Figs 7B, 8E and 8F, S7A and S7B Fig). However, we cannot rule out the possibility that some other signaling pathways or p53 status (such as modification or subcellular distribution) [74] might also been affected upon polyQ-expanded ATX-3 expression, which led to the change of the percentage of cells undergoing apoptosis and necrosis.

We first used zebrafish as a model vertebrate to test the influence of polyQ expansion on the neuronal cell death in vivo. It is intriguing to note that injection of *ATX-3* mRNA into the zebrafish embryos led to p53-dependent apoptosis, which occurred mainly in the central nervous system of zebrafish at early development stage (24 hpf). However, the polyQ-expanded ATX-3 induced progressive severe p53-dependent neurodegeneration in the central nervous system of zebrafish, suggesting that it caused other kinds of p53-mediated neural cell death besides apoptosis, too.

By generating a lentiviral-based in vivo MJD genetic model in p53^+/+^ and p53^-/-^ mice, our in situ detection of two apoptotic markers (TUNEL and active caspase-3) and one necrosis marker (RIP1) data have provided convincing evidences that both enhanced apoptotic-like and non-apoptotic cell death are observed in the SNpc and striatum of ATX-3^exp^ (80Q)-expressed neurons in p53^+/+^ mice brains but not in p53^-/-^ mice brains (Fig 8E and 8F, S7A–S7C Fig). Meanwhile, significantly more p53-positive cells were detected in ATX-3^exp^ (80Q)-expressed mouse brains sections (Fig 8A and 8B, S6C and S6D Fig). These data supported an idea that, due to enhanced interaction to p53 and up-regulation of p53, polyQ-expanded ATX-3 led to an increased p53-dependent neuronal cell death (including both early apoptotic and late apoptotic/necrotic manner). All together, the aforementioned studies and our results provide consistent evidences for the involvement of p53 in SCA3 pathogenesis, and the activation of the p53 pathway likely triggers neuronal dysfunction and eventually neuronal cell death in SCA3.

In conclusion, by identifying p53 as a new substrate of ATX-3, our study not only reveals a physiological function of ATX-3 and a new mechanism of p53 regulation but also establishes a novel molecular link between disease mutant ATX-3 and p53-mediated neurodegeneration, which sheds light on the molecular pathogenic mechanisms in SCA3.

## Materials and Methods

### Ethics Statement

Our work involving zebrafish and mouse experiments was in full compliance with the Regulations for the Care and Use of Laboratory Animals by the Ministry of Science and Technology of China and with the Institute of Zoology's Guidelines for the Care and Use of Laboratory Animals. The experimental protocols was approved by the Animal Care and Use Committee at the Institute of Zoology, Chinese Academy of Sciences (Permission Number: IOZ-13048).

### Cell Culture, Fish Embryos, Mice, Plasmids, and Antibodies

ATX-3^+/+^ and ATX-3^-/-^ MEFs, HEK293T, A549, RKO, and HeLa cells were cultured in DMEM supplemented with 10% FBS. U2OS, HCT116 p53^+/+^, and HCT116 p53^-/-^ cells were cultured in McCoy’s 5A supplemented with 10% FBS. *WT* zebrafish embryos were obtained from natural matings of zebrafish Tuebingen strain. *Tg(HuC*:*EGFP)* transgenic fish embryos express EGFP in the post-mitotic thalamic neurons. Homozygous *p53(M214K)* mutant fish line carrying a loss-of-function *p53* point mutation was kindly provided by Prof. Jinrong Peng at College of Life Sciences, Zhejiang University. Embryos were raised in Holtfreter’s solution at 28.5°C and staged by morphology as described [75]. *P53* +/- mice were obtained from Jackson Laboratory. *P53*+/- mice were intercrossed to get *P53*-/- mice and *P53*+/- mice. For genotyping the p53 locus, primers X7 (5′-TAT ACT CAG AGC CGG CCT-3′), NEO19 (5′-CAT TCA GGA CAT AGC GTT GG-3′), and X6.5 (5′-ACA GCG TGG TGG TAC CTT AT-3′) were used as described previously [76]. Four-wk-old mice were used. ATX-3 was cloned into pCS2-Flag, p3×Flag-CMV26-Myc, pGEX-4T-1, and pET-28a vectors. p53 was cloned into pCMV-HA, pCMV-Flag, pGEX-4T-1, and pET-28a vectors. ATX-3 mutants were generated by site-directed mutagenesis (Stratagene). Anti-ATX-3 (1H9) was purchased from Merck. Anti-p53 (DO-1, PAb1620, and pAb421) and anti-Bax were purchased from Calbiochem. Anti-p53 (FL393), anti-Ub, anti-p21, anti-Cyclin B1, and anti-PARP1 were purchased from SantaCruz. Anti-Flag (M2) was purchased from Sigma. Anti-His was purchased from Abmart. Anti-HA was purchased from Covance. Anti-Puma, anti-p17 specific caspase 3, anti-RIP1, and anti-HMGB1 were purchased from ProteinTech. Anti-GFP was purchased from Thermo Fisher Scientific Inc. Anti-DARPP-32 and anti-TH antibodies were purchased from Cell Signaling.

### Mass Spectrometry

3×Flag-ATX-3 transiently expressed in HEK293T cells was immunoprecipitated with anti-Flag M2 affinity gel and eluted with 3×Flag peptide (Sigma). Eluted proteins were identified with a gel-based liquid chromatography-tandem mass spectrometry (Gel-LC-MS/MS) approach and ion trap mass spectrometry (LTQ; Thermo Electron). A Mascot database search and the Scaffold program (Proteome Software) were used to visualize and validate results.

### Coimmunoprecipitation Assay

Cells were lysed with NETN buffer (20 mM Tris-HCl, pH 8.0, 100 mM NaCl, 1 mM EDTA, 0.5% Nonidet P-40) containing 1 mM Na_3_VO_4_, 10 mM NaF, 10 mM NEMi, and a cocktail of protease inhibitors. Whole cell lysates obtained by centrifugation were incubated with primary antibody overnight at 4°C. Protein A/G PLUS-Agarose beads (Santa Cruz) were then added and incubated for 2 h at 4°C. The immunocomplexes were then washed with NETN buffer for six times and separated by SDS-PAGE. Immunoblotting was performed following standard procedures.

### In Vitro Pull-Down Assays

GST and His fusion proteins were expressed in *Escherichia coli* strain BL21 (DE3) and affinity-purified using Glutathione-Sepharose 4B beads (GE Healthcare) or Ni-NTA Agarose (Qiagen), respectively, according to the manufacturer's instructions. For in vitro binding assays, bead-immobilized GST proteins were incubated with purified His proteins or with cell lysates in assay buffer at 4°C for 3 h followed by extensive washing. The bound proteins were separated by SDS-PAGE and analyzed by western blot with indicated antibodies.

### In Vitro Deubiquitination Assay

For the in vitro deubiquitination assays, the ubiquitinated p53 protein was incubated with recombinant GST-ATX-3 (100 ng) or the same amount of other indicated proteins in a deubiquitination buffer (50 mM Tris-HCl pH 8.0, 50 mM NaCl, 1 mM EDTA, 10 mM DTT, 5% glycerol) for 2 h at 37°C. Western blot was performed to detect p53 ubiquitination. GST controls were included in all DUB assays. The in vitro p53 ubiquitination assays were conducted in a total of 20 μl reaction buffer containing recombinant p53 (20 ng), Mdm2 (100 ng), UbE1 (0.025 μM, Boston Biochem), UbcH5 (0.4 μM, Biomol), Ub (40 μM, Boston Biochem), 50 mM Tris-HCl (pH 7.5), 5 mM MgCl_2_, 2 mM ATP, and 2 mM DTT in the absence or presence of varying amount of bacterial His-ATX-3 at 37°C for 2 h. The reactions were stopped by adding SDS sample buffer followed by immunoblotting with anti-p53 antibodies.

### Detection of Ubiquitination Levels of p53 In Vivo

The cells were treated with proteasome inhibitor MG132 (20 μM) for 4 h and then lysed in NETN lysis buffer with mild sonication. p53 was immunoprecipitated from the cell extract and subsequently resolved by SDS-PAGE and analyzed by western blot. For the preparation of a large amount of ubiquitinated p53 as the substrate for the deubiquitination assay in vitro, HEK293T cells were transfected together with the Flag-p53, HA-Ub, and Mdm2 expression vectors. After treatment as described above, the ubiquitinated p53 was purified from the cell extracts with anti-Flag M2 beads. After extensive washing, the proteins were eluted with Flag peptides (Sigma).

### Measurement of p53 Half-Life

For protein half-life assays, 20 μg/ml CHX was added to cell cultures to block protein synthesis. Cells were collected at indicated time points, and protein levels were measured by western blot. The relative intensities of the bands were determined by densitometry analyses using Photoshop 7.0 software (Adobe). The half-lives of proteins were calculated from three independent experiments. To determine which degradation pathway was involved, 20 μg/ml CHX was added for the indicated intervals in the presence of MG132 (20 μM), NH_4_Cl (20 mM), or 3-MA (5 mM).

### Transfection and Reporter Assays

ATX-3^+/+^ and ATX-3^-/-^ MEF cells were co-transfected with the indicated reporter constructs PG13 and the internal control Renilla luciferase pRL-null (pRL-CMV, Promega) at a ratio of 8:1 using PEI. Luciferase assays were performed using a dual-luciferase reporter assay system (E1910, Promega) according to the instructions of the manufacturer. Data were normalized for activity of Renilla luciferase to account for transfection efficiency. The assays were performed in duplicate, and data represent the average of five independent experiments.

### Flow Cytometry

For the flow cytometric analysis of cell cycle with PI DNA staining, the cells were harvested and washed once with PBS, followed by fixation in cold 70% ethanol at 4°C overnight. Then the cells were washed twice with PBS and treated with ribonuclease. Two hundred μl of PI was added before flow cytometry analysis. Apoptosis was assessed by using Becton—Dickinson FACScan flow cytometer according to the manufacturer’s instructions. Cells were treated with or without 1 μM CPT (a topoisomerase I inhibitor) for 24 h. Cells were collected and washed once with PBS, followed by incubation in annexin V (A13201; Invitrogen) solution in dark at room temperature for 15 min and 10 μl of PI in annexin V binding buffer. Flow cytometry analysis was carried out within 1 h. Data analysis was performed with CellQuest software. The numbers of apoptotic cells that are positive for annexin V staining (positive and negative for PI staining) were counted as a proportion to the total number of gated cells and expressed as percent of apoptotic cells in a histogram. Early apoptotic cells are positive for annexin V staining and negative for PI staining, whereas late apoptotic/necrotic cells are positive for annexin V and PI staining due to a loss of plasma membrane integrity.

### RNA Extraction and Quantitative Real Time PCR Analysis

Total RNA was extracted from HCT116 or MEF cells using TRIzol (Invitrogen), and 1.5 μg of total RNA was used to prepare the first-strand cDNA using the SuperScript II polymerase (Invitrogen). Quantitative real-time PCR reactions were carried out in triplicate on a Thermal Cycler using SYBR Green dye to measure amplification. Relative mRNA levels of each gene shown were normalized to the expression of the housekeeping genes GAPDH.

### Nuclear DNA Staining

Normal, necrotic, and apoptotic cells were observed under fluorescence microscopy. Cells were fixed by 4% paraformaldehyde in PBS, and their nuclear DNA was stained with Hoechst-33342 for detection of necrosis and apoptosis by morphological features, according to Gschwind and Huber [77].

### Apoptosis Analyses in Zebrafish

mRNA of FL and various ATX-3 mutants were in vitro synthesized from corresponding linearized plasmids using mMESSAGE mMACHINE Kit (Ambion). Digoxigenin-UTP-labeled antisense RNA probes were transcribed in vitro using MEGAscript Kit (Ambion) according to the manufacturer’s instructions. Microinjection and whole-mount in situ hybridization were performed as before [78–81]. Apoptosis were determined by TUNEL assay [82]. *WT* and *p53 mutant* zebrafish embryos were injected with object mRNA at one-cell stage and then harvested at 24 hpf for TUNEL labeling using In Situ Cell Death Detection Kit, TMR red (12156792910, Roche) according to the manufacturer’s instruction. *Tg(HuC*:*EGFP)* zebrafish embryos were fixed and stained with anti-GFP antibody. TUNEL-positive cells were imaged by confocal microscopy. Images were analyzed with ImageJ software. Significance was analyzed using the unpaired *t* test.

### Production of Lentiviral Vectors

Lentiviral-EGFP vectors encoding human WT ATX-3 or mutant ATX-3, including ATX-3-80Q and ATX-3-C14A, were produced in 293T cells with a three-plasmid system. The lentiviral particles were re-suspended in artificial cerebrospinal fluid. Viral stocks were stored at -80°C.

### Stereotactic Injection

Mice were anesthetized with 1% pentobarbital sodium and then placed into a stereotactic frame. Concentrated viral stocks were thawed on ice. L were stereotaxically injected into the striatum at the following coordinates: anterior-posterior: + 0.6 mm from the bregma; mediolateral: -2.5 mm from midline; and dorsoventral: -3.2 mm below surface of the dura; tooth bar: 0. LV were stereotaxically injected into the SNpc at the following coordinates: anterior-posterior: -3.8 mm from the bregma; mediolateral: -2.1 mm from midline; and dorsoventral: -4.5 mm below surface of the dura; tooth bar: 0. *P53*^+/+^ and *P53*^-/-^ mice received a single injection of lentivirus in each side: left hemisphere (LV-GFP) and right hemisphere (ATX-3-WT, ATX-3-80Q, or ATX-3-C14A). Mice were kept in their home cages for 8 wk before being killed for immunostaining analysis.

### Neuropathological Analysis

Mice were perfused transcardially with 0.1 M phosphate buffered saline followed by fixation with 4% paraformaldehyde. Serial coronal sections were cut through the entire striatum and SNpc at 25 μm. Free-floating cryosections from injected mice were blocked in PBS/0.3% TritonX-100 containing 10% normal donkey serum (Gibco) and then incubated overnight at 4°C with the following primary antibodies: a rabbit polyclonal anti- DARPP-32 antibody (Cell Signaling, 1:200); a rabbit polyclonal anti-TH antibody (Cell Signaling, 1:200); a mouse monoclonal anti-p53 antibody, clone PAb1620 (1:50; Millipore); a rabbit polyclonal anti-RIP1-specific antibody (1:50, Proteintech); and a rabbit polyclonal anti-caspase 3, p17-specific antibody (1:50, Proteintech). Sections were washed in PBS and then incubated with the corresponding secondary antibodies coupled to fluorophores (Molecular Probes) for 1 h at 37°C. TUNEL analysis was performed using cryosections with the ApopTag-fluorescein in situ apoptosis detection kit (Chemicon) according the manufacturer's instruction. Apoptotic cells were quantified, and apoptotic indices were calculated by computer-assisted image analysis following identification of apoptotic cells by morphological analysis, the TUNEL assay, or activated caspase-3 immunostaining. Imaging was done by total internal reflection fluorescence microscope and three-dimensional structured illumination microscopy. Cell counts were determined from anatomically matched sections from each of the animals, and three animals were used for cell counts.

## Supporting Information

S1 Fig Ataxin-3 interacts with p53. (A) 293T cells were transfected with the indicated Flag-ataxin-3 full-length or its mutants. Cell lysates were immunoprecipitated by M2 beads, and the bound proteins were analyzed by immunoblotting with anti-p53 polyclonal antibody. (B and C) GST-pull down assay was performed with GST or the indicated GST-fused proteins and the whole cell extract of A549. GST proteins were stained by Ponceau S, and the pulled down proteins were analyzed by immunoblotting with anti-p53 polyclonal antibody. (D) GST-pull down assay was performed with the GST-Ub chains and the whole cell extract of 293T transiently transfected with indicated Flag-tagged ataxin-3 and its mutants. GST-Ub was stained by Ponceau S, and the pulled down proteins were analyzed by immunoblotting with anti-Flag antibody. (E) Effects of ataxin-3 gene knockout on p53 protein levels in primary MEFs. Ablation of ataxin-3 gene caused significantly decreased p53 levels in KO primary MEFs. Each lane represents one primary MEF culture from one fetus. We should note here that, the p53 protein levels in primary KO MEFs were gradually increasing with the passages number increase during immortalization, suggesting a compensation for the absence of ataxin-3 may occur in KO MEF cells during immortalization.
(TIF)

S2 Fig Ataxin-3 regulates p53-responsive gene expression. (A, B) qRT-PCR (A) and western blot (B) analysis of p53 downstream targets in ataxin-3+/+ and ataxin-3-/- MEF cells, transfected with indicated plasmids. Relative mRNA levels were normalized to GAPDH (mean ± SEM; n = 3 or 4). * denotes P<0.05, and ** denotes P<0.01. (C-F) qRT-PCR (C and D) and western blot (E and F) analysis of p53 downstream targets in HCT116 p53+/+ and HCT116 p53-/- control and ataxin-3 stably knockdown cells, transiently transfected with empty vector or plasmid encoding Flag-ataxin-3-C14A (C and E) or Flag-ataxin-3-S/A (D and F). Relative mRNA levels were normalized to GAPDH (mean ± SEM; n = 3 or 4). * denotes P<0.05, and ** denotes P<0.01. (G) HCT116 p53+/+ and HCT116 p53-/- ataxin-3-stably knockdown cells were fixed, stained with PI, and analyzed by flow cytometry. The data represent the mean ± SEM for three individual experiments. * denotes P<0.05. Underlying data are shown in S1 Data.
(TIF)

S3 Fig Ataxin-3 expression-induced cell death occurs in cells and in HuC positive brain regions in zebrafish. (A) Flow cytometry analysis using Annexin V-FITC/PI staining in HCT116 p53+/+ cells. (B and C) Dorsal views with anterior to the top of Tg(HuC:EGFP) embryos. Colocalization of HuC:EGFP (green) and TUNEL positive foci (red) in the telencephalon region (B) and diencephalon/hindbrain (C). Tg(HuC:EGFP) transgenic embryos uninjected control (UIC) or injected with ataxin-3 were collected for TUNEL staining at 24 hpf. Scale bars, 20 μm for B and 50 μm for C.
(TIF)

S4 Fig PolyQ-expanded ataxin-3 regulates p53 function and stability. (A and B) qRT-PCR (A) and western blot (B) analysis of p53 downstream targets in HCT116 p53+/+ and HCT116 p53-/- control and ataxin-3 stably knockdown cells, transiently transfected with empty vector or plasmid encoding Flag-ataxin-3-80Q. Relative mRNA levels were normalized to GAPDH (mean ± SEM; n = 3). * denotes P<0.05. (C and D) HCT116 cells (C) and RKO cells (D) transiently transfected with Flag-ataxin-3-80Q (ATX-3-80Q) or Flag-ataxin-3 (ATX-3-WT) were treated with 20 μg/ml CHX for the indicated times, and then were subjected to immunoblotting for p53, Flag and β-actin (left). p53 protein levels were quantified and normalized to β-actin. The data is representative of one of the three independent experiments (Right). (E) Effects of ectopic expressions of polyQ expanded ataxin-3 and ataxin-3-WT on p53 protein levels in different cell lines. Cells expressing Flag-ataxin-3-80Q (ATX-3-80Q) or Flag-ataxin-3 (ATX-3-WT) as well as primary WT and ataxin-3-84Q MEFs were lysed and then subjected to immunoblotting with indicated antibodies. Expressions of polyQ expanded ataxin-3 led to significantly higher p53 protein levels in RKO, 293T, and primary MEF cells. (F) Western blot analysis of p53 downstream targets in RKO cells. RKO cells transfected with empty vector or plasmid encoding Flag-ataxin-3-WT or Flag-ataxin-3-80Q were collected, lysed and then subjected to immunoblotting with indicated antibodies.
(TIF)

S5 Fig PolyQ expansion in ataxin-3 induces p53-dependent neurodegeneration in zebrafish. (A) Normal, apoptotic and late apoptotic/necrotic cells were observed by staining of nuclear DNA with Hoechst-33342 under fluorescence microscopy. HCT116 p53+/+ and HCT116 p53-/- cells transiently transfected with Flag-ataxin-3-80Q or Flag-ataxin-3 were left untreated (upper) or treated with 1μM of CPT (lower) for 24h. Cells were fixed with 4% paraformaldehyde in PBS and their nuclear DNA was stained with Hoechst-33342 for detection of necrosis and apoptosis by morphological features. (B) Whole-mount in situ hybridization analyses of the midbrain neural marker otx2 in uninjected control (UIC) or normal ataxin-3 (WT) or ataxin-3exp (80Q) mRNA-injected WT (left) and p53 mutant (right) zebrafish embryos at 24, 48, and 72 hpf. Embryos were shown in dorsal views with anterior to the left. The ratio of embryos with the representative phenotypes was indicated. Both WT and 80Q mRNA injections resulted in obvious otx2 signal decreases (indicated by arrowheads) in wild-type but not p53 mutant zebrafishes, with more profound otx2 signal loss in 80Q mRNA injections. MB denotes midbrain. (C) Whole-mount in situ hybridization analyses of the central nervous system marker ngn1 in UIC or normal ataxin-3 (WT) or ataxin-3exp (80Q) mRNA injected-WT (upper) and p53 mutant (lower) zebrafish embryos at 24, 48, and 72 hpf. Embryos were shown in both lateral views (left) and dorsal views (right) with anterior to the left. The ratio of embryos with the representative phenotypes was indicated. TE denotes telencephalon; MB denotes midbrain; HB denotes hindbrain.
(TIF)

S6 Fig PolyQ expanded ataxin-3 caused p53-dependent neurodegeneration in the striatum of MJD transgenic mice. (A-B) Imaging (A) and western blot analysis (B) of 293T cells infected with lentiviral vectors encoding ATX-3-WT or mutant ATX-3 (ATX-3-80Q or ATX-3-C14A) containing a EGFP tag at 5 days post-infection. The membrane was probed with anti-ataxin-3 (1H9) antibody. (C and E) Immunostaining of DARPP32 (left panel) and p53 (right panel) in the striatum of p53+/+ (C) and p53-/- (E) mice. Scale bar = 250 μm. (D) Quantitative analysis of the number of DARPP32- and p53- positive neurons in p53+/+ mouse brains injected in the striatum with LV. Values are presented as means ± S.D. of 3 mice per group and 1 or 2 sections per mouse. * denotes P<0.05, ** denotes P<0.01, *** denotes P<0.001. (F) Quantitative analysis of the number of DARPP32-positive neurons in p53-/- mouse brains injected in the striatum with LV. Values are presented as means ± S.D. No statistical significance was observed among these groups. Underlying data are shown in S1 Data.
(TIF)

S7 Fig Assessment of neuronal cell death in the striatum and SNpc of p53+/+ and p53-/- mice. (A) Brain sections through the striatum were stained using the TUNEL method or anti-activated caspase-3 antibody or anti-RIP1 antibody (red panel). Images shown in this figure are representative of three repeated experiments. Scale bar = 250 μm. (B) Quantitation of the percentage of TUNEL-positive signals or the percentage of cells positive for activated caspase-3 or RIP1 in the population of EGFP-positive neurons in p53+/+ mice. Values are presented as means ± S.D. n = 3. * denotes P<0.05. (C) Immunostaining of TH and RIP1 in the SNpc of p53+/+ and p53-/- mice at a higher magnification. Scale bar = 50 μm. Underlying data are shown in S1 Data.
(TIF)

S1 Data (XLS)

## Abbreviations

ATX-3: ataxin-3
CAG: cytosine-adenine-guanine
CHX: cycloheximide
CPT: camptothecin
DARPP-32: dopamine- and cyclic AMP-regulated neuronal phosphoprotein
DOX: doxycycline
DUB: deubiquitinating enzyme
EGFP: enhanced green fluorescent protein
FACS: fluorescence-activated cell sorting
FL: full-length
GAPDH: glyceraldehydes-3-phosphate dehydrogenase
GFP: green fluorescent protein
GST: glutathione S-transferase
HB: hindbrain
HMGB1: High Mobility Group Box 1
hpf: hours post fertilization
KO: knockout
LV: lentiviral vectors
3-MA: 3-methyladenine
MB: midbrain
MEF: mouse embryonic fibroblast
MJD: Machado–Joseph disease
NEMi: N-ethylmaleimide
Ni-NTA: nickel-nitrilotriacetic acid
OTU: otubain
PARP1: poly (ADP-ribose) polymerase
PI: propidium iodide
PolyQ: polyglutamine
qRT-PCR: quantitative reverse transcription-polymerase chain reaction
RIP1: receptor-interacting serine/threonine protein kinases 1
SCA3: spinocerebellar ataxia type 3
shRNA: short hairpin RNA
SNpc: substantia nigra pars compacta
TE: telencephalon
TH: tyrosine hydroxylase
Ub: ubiquitin
UIC: uninjected control
UIMs: ubiquitin-interacting motifs
USP: ubiquitin-specific protease
VCP: valosin-containing protein
WT: wild-type
