# Supplementary figures and images for: The Machado–Joseph Disease Deubiquitinase Ataxin-3 Regulates the Stability and Apoptotic Function of p53
Source: PLoS Biol. 2016 Nov 16;14(11):e2000733. doi: 10.1371/journal.pbio.2000733 (PMC5112960; doi:10.1371/journal.pbio.2000733)

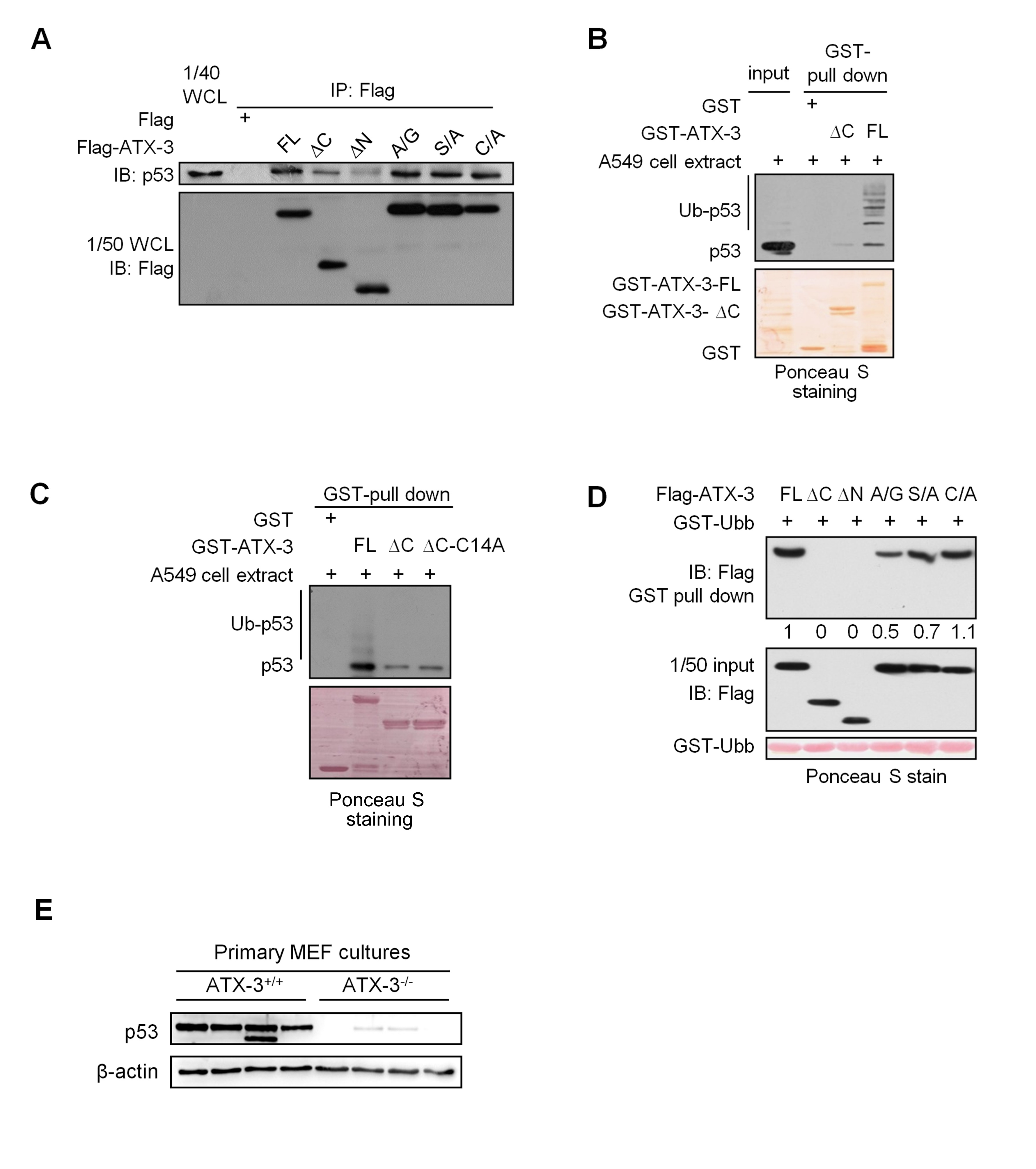

Supplement: S1 Fig — (A) 293T cells were transfected with the indicated Flag-ataxin-3 full-length or its mutants. Cell lysates were immunoprecipitated by M2 beads, and the bound proteins were analyzed by immunoblotting with anti-p53 polyclonal antibody. (B and C) GST-pull down assay was performed with GST or the indicated GST-fused proteins and the whole cell extract of A549. GST proteins were stained by Ponceau S, and the pulled down proteins were analyzed by immunoblotting with anti-p53 polyclonal antibody. (D) GST-pull down assay was performed with the GST-Ub chains and the whole cell extract of 293T transiently transfected with indicated Flag-tagged ataxin-3 and its mutants. GST-Ub was stained by Ponceau S, and the pulled down proteins were analyzed by immunoblotting with anti-Flag antibody. (E) Effects of ataxin-3 gene knockout on p53 protein levels in primary MEFs. Ablation of ataxin-3 gene caused significantly decreased p53 levels in KO primary MEFs. Each lane represents one primary MEF culture from one fetus. We should note here that, the p53 protein levels in primary KO MEFs were gradually increasing with the passages number increase during immortalization, suggesting a compensation for the absence of ataxin-3 may occur in KO MEF cells during immortalization. (TIF) [file pbio.2000733.s001.tif]

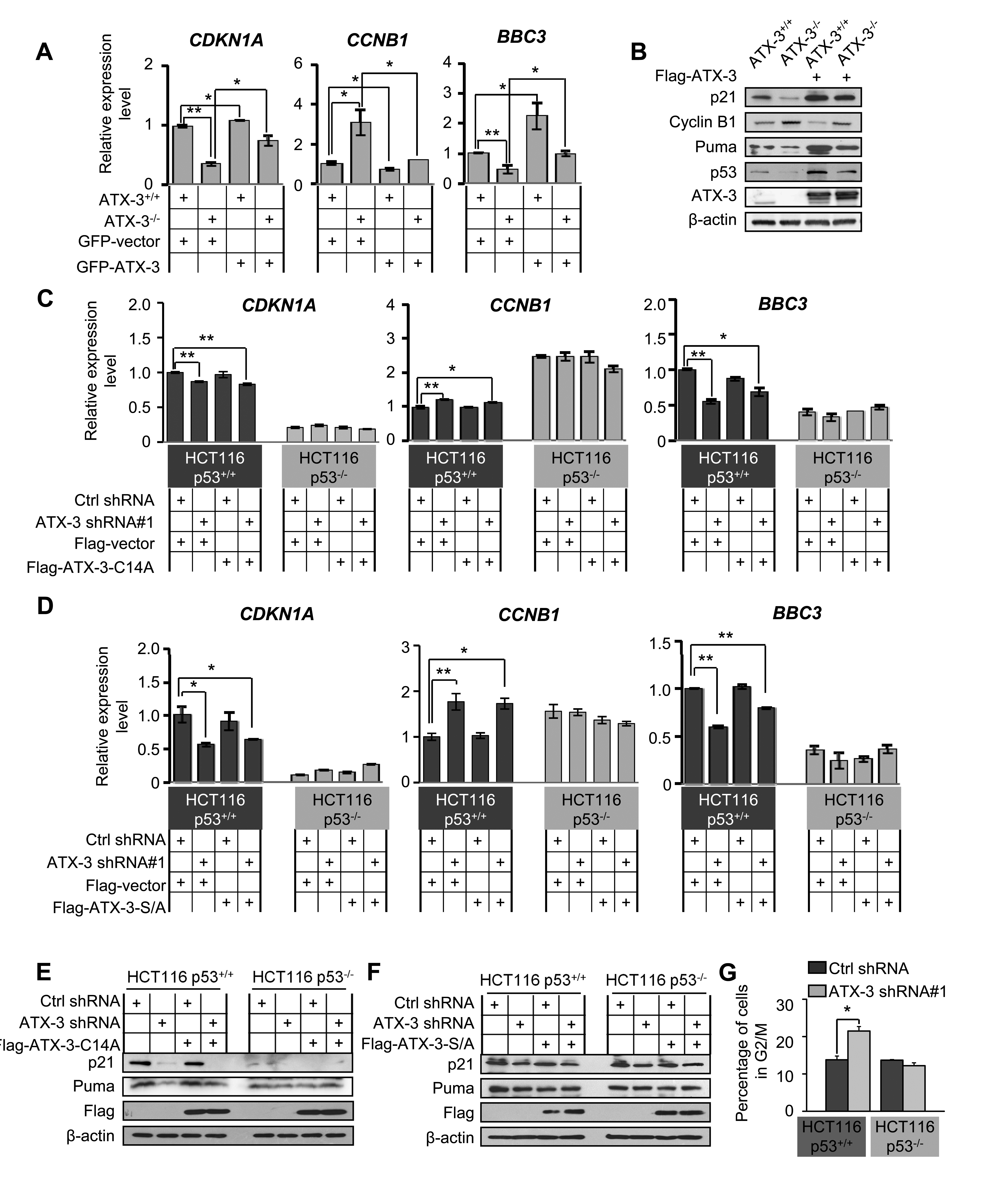

Supplement: S2 Fig — (A, B) qRT-PCR (A) and western blot (B) analysis of p53 downstream targets in ataxin-3+/+ and ataxin-3-/- MEF cells, transfected with indicated plasmids. Relative mRNA levels were normalized to GAPDH (mean ± SEM; n = 3 or 4). * denotes P<0.05, and ** denotes P<0.01. (C-F) qRT-PCR (C and D) and western blot (E and F) analysis of p53 downstream targets in HCT116 p53+/+ and HCT116 p53-/- control and ataxin-3 stably knockdown cells, transiently transfected with empty vector or plasmid encoding Flag-ataxin-3-C14A (C and E) or Flag-ataxin-3-S/A (D and F). Relative mRNA levels were normalized to GAPDH (mean ± SEM; n = 3 or 4). * denotes P<0.05, and ** denotes P<0.01. (G) HCT116 p53+/+ and HCT116 p53-/- ataxin-3-stably knockdown cells were fixed, stained with PI, and analyzed by flow cytometry. The data represent the mean ± SEM for three individual experiments. * denotes P<0.05. Underlying data are shown in S1 Data. (TIF) [file pbio.2000733.s002.tif]

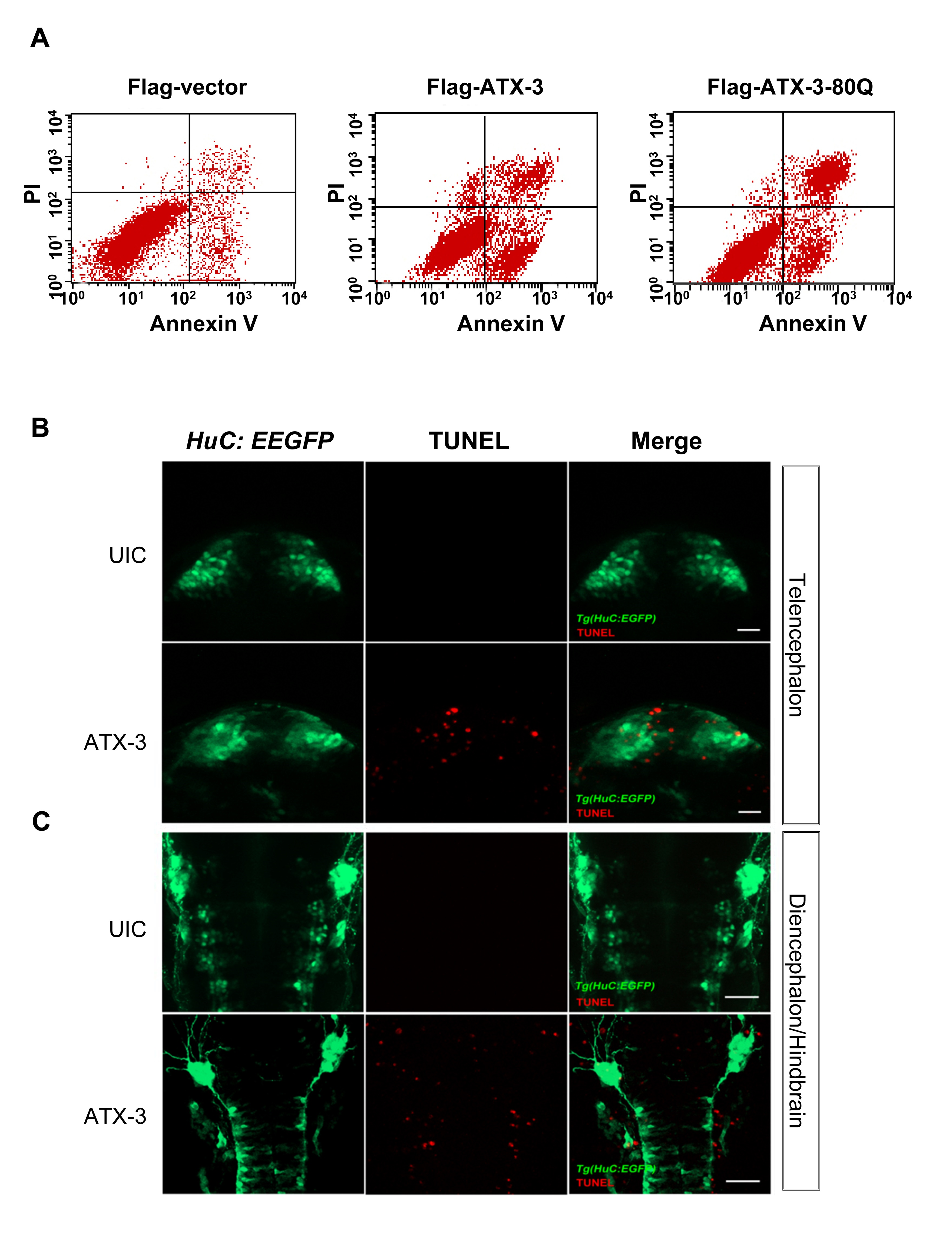

Supplement: S3 Fig — (A) Flow cytometry analysis using Annexin V-FITC/PI staining in HCT116 p53+/+ cells. (B and C) Dorsal views with anterior to the top of Tg(HuC:EGFP) embryos. Colocalization of HuC:EGFP (green) and TUNEL positive foci (red) in the telencephalon region (B) and diencephalon/hindbrain (C). Tg(HuC:EGFP) transgenic embryos uninjected control (UIC) or injected with ataxin-3 were collected for TUNEL staining at 24 hpf. Scale bars, 20 μm for B and 50 μm for C. (TIF) [file pbio.2000733.s003.tif]

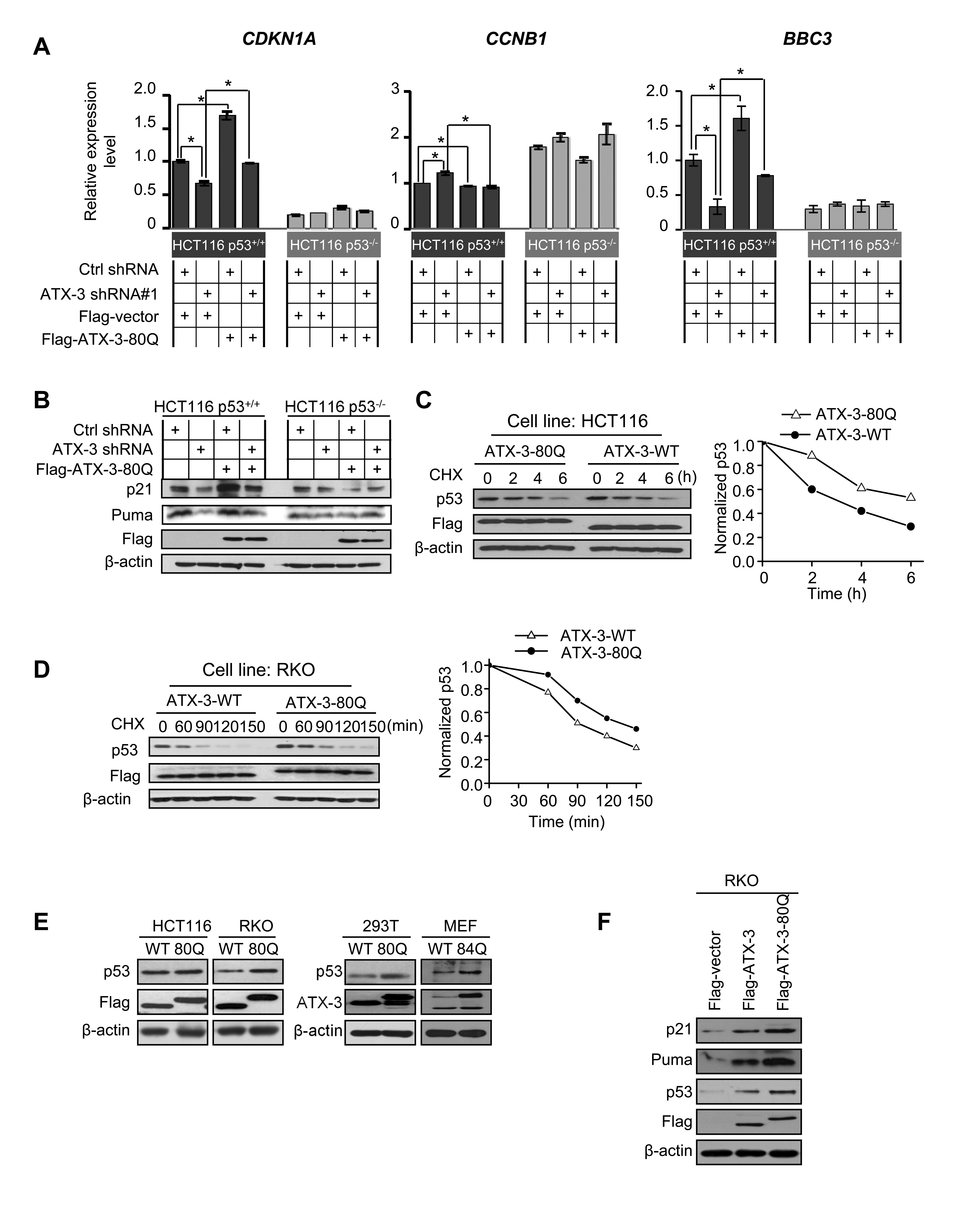

Supplement: S4 Fig — (A and B) qRT-PCR (A) and western blot (B) analysis of p53 downstream targets in HCT116 p53+/+ and HCT116 p53-/- control and ataxin-3 stably knockdown cells, transiently transfected with empty vector or plasmid encoding Flag-ataxin-3-80Q. Relative mRNA levels were normalized to GAPDH (mean ± SEM; n = 3). * denotes P<0.05. (C and D) HCT116 cells (C) and RKO cells (D) transiently transfected with Flag-ataxin-3-80Q (ATX-3-80Q) or Flag-ataxin-3 (ATX-3-WT) were treated with 20 μg/ml CHX for the indicated times, and then were subjected to immunoblotting for p53, Flag and β-actin (left). p53 protein levels were quantified and normalized to β-actin. The data is representative of one of the three independent experiments (Right). (E) Effects of ectopic expressions of polyQ expanded ataxin-3 and ataxin-3-WT on p53 protein levels in different cell lines. Cells expressing Flag-ataxin-3-80Q (ATX-3-80Q) or Flag-ataxin-3 (ATX-3-WT) as well as primary WT and ataxin-3-84Q MEFs were lysed and then subjected to immunoblotting with indicated antibodies. Expressions of polyQ expanded ataxin-3 led to significantly higher p53 protein levels in RKO, 293T, and primary MEF cells. (F) Western blot analysis of p53 downstream targets in RKO cells. RKO cells transfected with empty vector or plasmid encoding Flag-ataxin-3-WT or Flag-ataxin-3-80Q were collected, lysed and then subjected to immunoblotting with indicated antibodies. (TIF) [file pbio.2000733.s004.tif]

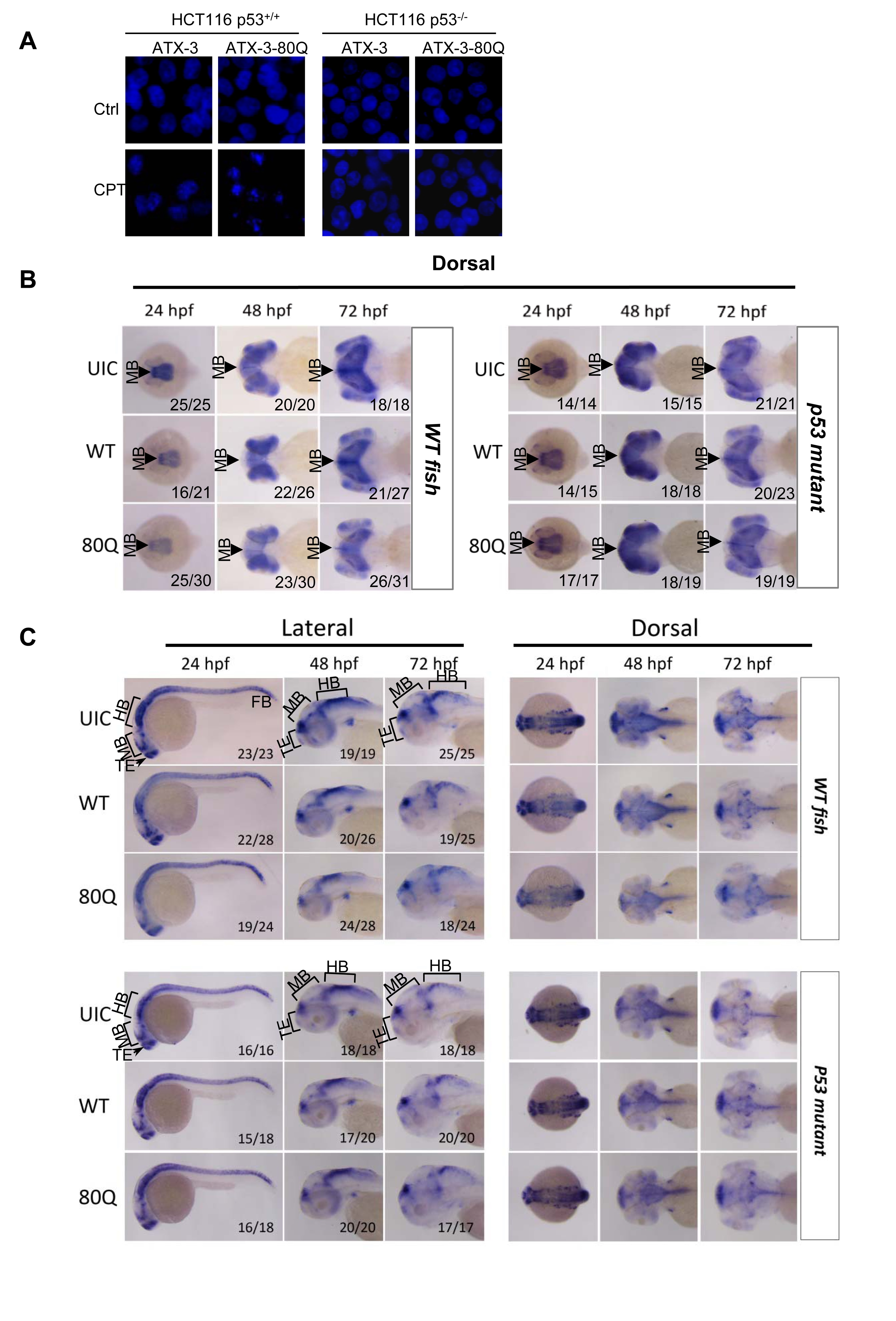

Supplement: S5 Fig — (A) Normal, apoptotic and late apoptotic/necrotic cells were observed by staining of nuclear DNA with Hoechst-33342 under fluorescence microscopy. HCT116 p53+/+ and HCT116 p53-/- cells transiently transfected with Flag-ataxin-3-80Q or Flag-ataxin-3 were left untreated (upper) or treated with 1μM of CPT (lower) for 24h. Cells were fixed with 4% paraformaldehyde in PBS and their nuclear DNA was stained with Hoechst-33342 for detection of necrosis and apoptosis by morphological features. (B) Whole-mount in situ hybridization analyses of the midbrain neural marker otx2 in uninjected control (UIC) or normal ataxin-3 (WT) or ataxin-3exp (80Q) mRNA-injected WT (left) and p53 mutant (right) zebrafish embryos at 24, 48, and 72 hpf. Embryos were shown in dorsal views with anterior to the left. The ratio of embryos with the representative phenotypes was indicated. Both WT and 80Q mRNA injections resulted in obvious otx2 signal decreases (indicated by arrowheads) in wild-type but not p53 mutant zebrafishes, with more profound otx2 signal loss in 80Q mRNA injections. MB denotes midbrain. (C) Whole-mount in situ hybridization analyses of the central nervous system marker ngn1 in UIC or normal ataxin-3 (WT) or ataxin-3exp (80Q) mRNA injected-WT (upper) and p53 mutant (lower) zebrafish embryos at 24, 48, and 72 hpf. Embryos were shown in both lateral views (left) and dorsal views (right) with anterior to the left. The ratio of embryos with the representative phenotypes was indicated. TE denotes telencephalon; MB denotes midbrain; HB denotes hindbrain. (TIF) [file pbio.2000733.s005.tif]

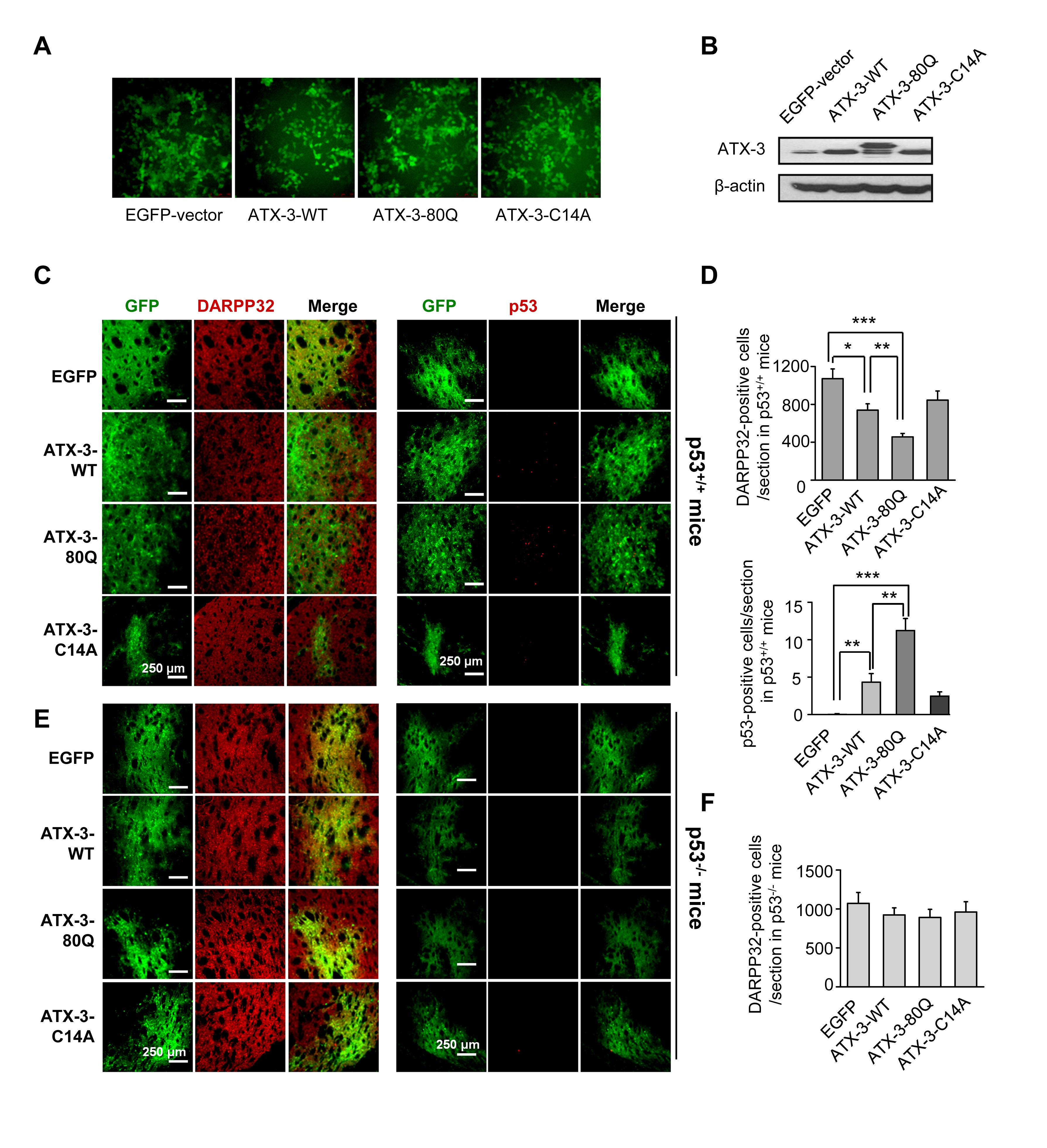

Supplement: S6 Fig — (A-B) Imaging (A) and western blot analysis (B) of 293T cells infected with lentiviral vectors encoding ATX-3-WT or mutant ATX-3 (ATX-3-80Q or ATX-3-C14A) containing a EGFP tag at 5 days post-infection. The membrane was probed with anti-ataxin-3 (1H9) antibody. (C and E) Immunostaining of DARPP32 (left panel) and p53 (right panel) in the striatum of p53+/+ (C) and p53-/- (E) mice. Scale bar = 250 μm. (D) Quantitative analysis of the number of DARPP32- and p53- positive neurons in p53+/+ mouse brains injected in the striatum with LV. Values are presented as means ± S.D. of 3 mice per group and 1 or 2 sections per mouse. * denotes P<0.05, ** denotes P<0.01, *** denotes P<0.001. (F) Quantitative analysis of the number of DARPP32-positive neurons in p53-/- mouse brains injected in the striatum with LV. Values are presented as means ± S.D. No statistical significance was observed among these groups. Underlying data are shown in S1 Data. (TIF) [file pbio.2000733.s006.tif]

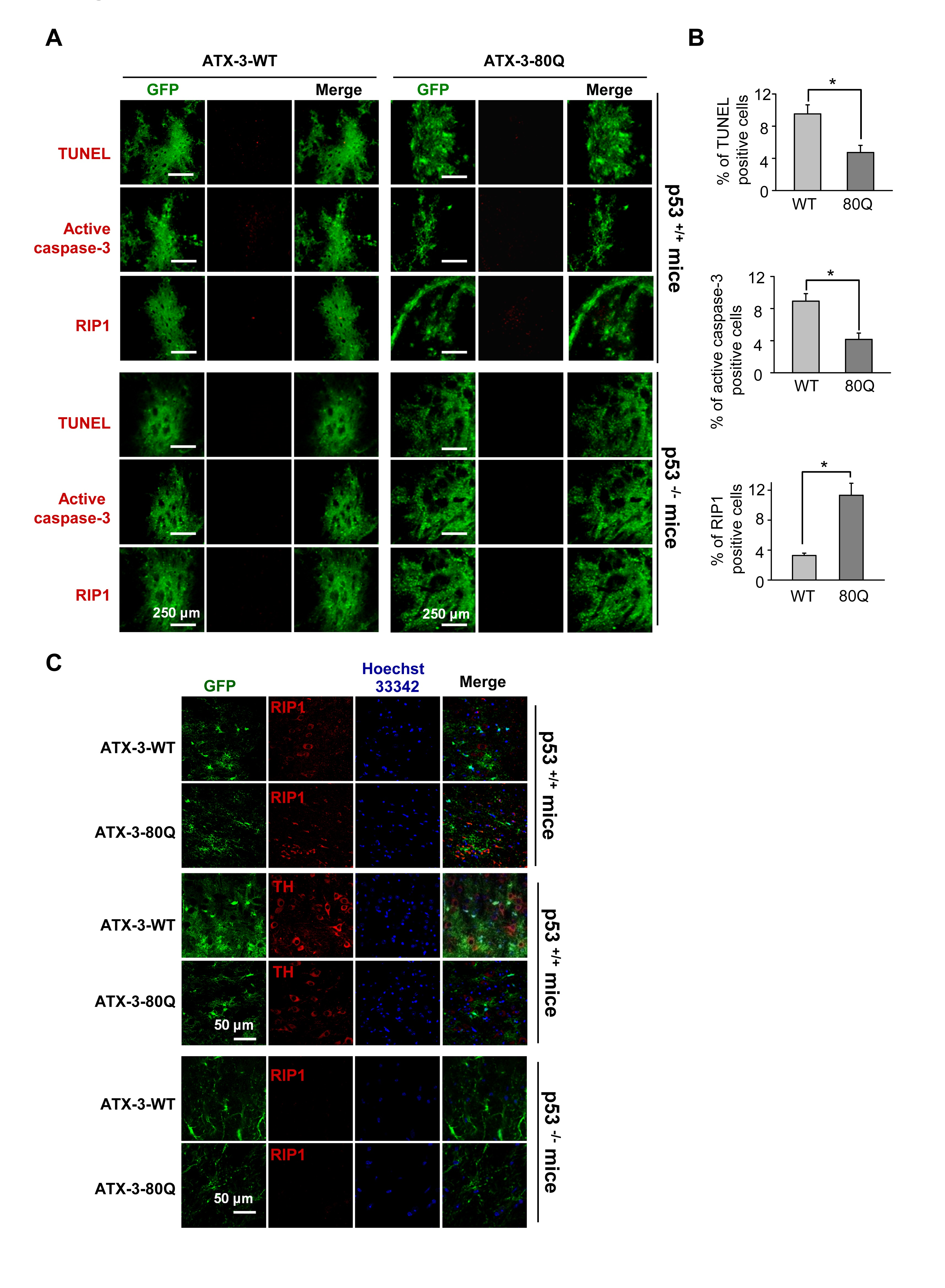

Supplement: S7 Fig — (A) Brain sections through the striatum were stained using the TUNEL method or anti-activated caspase-3 antibody or anti-RIP1 antibody (red panel). Images shown in this figure are representative of three repeated experiments. Scale bar = 250 μm. (B) Quantitation of the percentage of TUNEL-positive signals or the percentage of cells positive for activated caspase-3 or RIP1 in the population of EGFP-positive neurons in p53+/+ mice. Values are presented as means ± S.D. n = 3. * denotes P<0.05. (C) Immunostaining of TH and RIP1 in the SNpc of p53+/+ and p53-/- mice at a higher magnification. Scale bar = 50 μm. Underlying data are shown in S1 Data. (TIF) [file pbio.2000733.s007.tif]
